# The major‐effect quantitative trait locus *Fnl7.1* encodes a late embryogenesis abundant protein associated with fruit neck length in cucumber

**DOI:** 10.1111/pbi.13326

**Published:** 2020-01-24

**Authors:** Xuewen Xu, Chenxi Wei, Qianya Liu, Wenqing Qu, Xiaohua Qi, Qiang Xu, Xuehao Chen

**Affiliations:** ^1^ School of Horticulture and Plant Protection Yangzhou University Yangzhou Jiangsu China; ^2^ Joint International Research Laboratory of Agriculture and Agri‐Product Safety the Ministry of Education of China Yangzhou University Yangzhou Jiangsu China

**Keywords:** Cucumber, fruit neck length, QTL, fine genetic mapping, late embryogenesis abundant protein

## Abstract

Fruit neck length (FNL) is an important quality trait in cucumber because it directly affects its market value. However, its genetic basis remains largely unknown. We identified a candidate gene for FNL in cucumber using a next‐generation sequencing‐based bulked segregant analysis in F_2_ populations, derived from a cross between Jin5‐508 (long necked) and YN (short necked). A quantitative trait locus (QTL) on chromosome 7, *Fnl7.1*, was identified through a genome‐wide comparison of single nucleotide polymorphisms between long and short FNL F_2_ pools, and it was confirmed by traditional QTL mapping in multiple environments. Fine genetic mapping, sequences alignment and gene expression analysis revealed that *CsFnl7.1* was the most likely candidate *Fnl7.1* locus, which encodes a late embryogenesis abundant protein. The increased expression of *CsFnl7.*1 in long‐necked Jin5‐508 may be attributed to mutations in the promoter region upstream of the gene body. The function of *CsFnl7.1* in FNL control was confirmed by its overexpression in transgenic cucumbers. CsFnl7.1 regulates fruit neck development by modulating cell expansion. Probably, this is achieved through the direct protein–protein interactions between CsFnl7.1 and a dynamin‐related protein CsDRP6 and a germin‐like protein CsGLP1. Geographical distribution differences of the FNL phenotype were found among the different cucumber types. The East Asian and Eurasian cucumber accessions were highly enriched with the long‐necked and short‐necked phenotypes, respectively. A further phylogenetic analysis revealed that the *Fnl7.1* locus might have originated from India. Thus, these data support that the CsFnl7.1 has an important role in increasing cucumber FNL.

## Introduction

Cucumber (*Cucumis sativus* L., 2*n* = 2*x* = 14) is produced worldwide and is consumed fresh or processed. Fruit neck (also called ‘stalk’) is defined as the first part of the cucumber fruit, joining it with the pedicel. It has no internal placenta and no spines or tubercles on the surface (Fanourakis and Tzifaki, 1992). The fruit neck length (FNL) at harvest varies from 1 to 12 cm, accounting for as much as 35% of the total fruit length (Zhao *et al.*, 2015). Typically, FNL directly affects appearance because of the non‐uniform diameter along the fruit, and it produces an undesirable taste owing to the absence of fleshy tissue and the occurrence of bitterness (Che and Zhang, 2019; Fanourakis and Tzifaki, 1992). In addition, the neck is easily broken, resulting in damage during harvesting, packing and transport (Fanourakis and Tzifaki, 1992). Thus, the presence of the fruit neck constitutes an important quality issue in fresh cucumber markets. Typically, breeding for short‐necked varieties is more desirable. In north China premium‐grade fresh market cucumbers, the neck should account for less than 14.3% (1/7) of the total fruit length (Zhou *et al.*, 2005).

Studies on the genetic architecture of the FNL trait in cucumber are very limited. Gu *et al. *(1994) found that the additive genetic variance for FNL accounted for 97.9% of the total phenotypic variance, indicating the importance of genetic rather than environmental variability in the trait. Ma *et al. *(2010) reported on the inheritance of FNL in cucumber using the mixed major gene and polygene inheritance model. They found that the genetic mode E‐1 model, in which two additive, dominant and epistatic major genes and additive‐dominant polygenes are mixed, is the best‐fitting genetic model for the trait. QTL mapping studies have been unable to consistently determine the numbers and locations of QTLs. Wang *et al. *(2008) were the first to conduct QTL mapping to identify QTLs for FNL in cucumber. They detected one major‐effect QTL (*R* ^2^ = 18.5%) controlling FNL in F_2_ populations from a cross of the north China‐type cucumber ‘129’ with the European greenhouse‐type cucumber ‘Z3’. Using 130 F_2_ progeny derived from a cross between ‘S06’ (northern European type) with ‘S94’ (northern China type), Yuan *et al. *(2008) detected four QTLs for FNL, each of which accounted for 8.8–30.2% of the phenotypic variation. More recently, using 160 inbred recombinant lines developed from a cross between ‘931’ (north China type) with wild cucumber accession ‘PI183967’ (*C. sativus* var. *hardwickii*, CSH), Wang *et al. *(2014) detected four QTLs on chromosomes 3, 6 and 7. However, no genes or QTLs for the ability to form fruit neck have been cloned from these studies.

The objectives of this research were to conduct fine genetic mapping to identify candidate gene responsible for the control of neck length. We developed segregating populations (F_2_ and F_2:3_) from the cross between long‐necked parent Jin5‐508 (north China‐type cucumber) and short‐necked parent YN (a North American‐type cucumber). We conducted high‐throughput sequencing of two DNA bulks (QTL‐seq) selected from F_2_ plants with extreme FNLs. The identified QTL‐seq‐derived major‐effect QTL was validated by traditional QTL mapping approaches. A further segregating analysis, gene expression analysis, as well as a transgenic analysis in cucumber, confirmed *CsFnl7.1* as the candidate gene for the *Fnl* locus. We provide evidence of a promoter polymorphism being the main cis‐regulatory factor involved in the control of *CsFnl7.1* expression levels. We also examined the allelic diversity of this locus in natural cucumber populations, which revealed the origin and evolution of this gene. The results of this study have provided new insights into genetic control of FNL in cucumber.

## Experimental procedures

### Plant materials and phenotype collection

Jin5‐508 is an advanced self‐pollinating inbred cucumber line derived from Jinchun5 (a typical northern China‐type commercial inbred line) through self‐pollination. YN is a highly inbred (>S10) line developed from cultivar Yunv that has a white‐spine, round‐shape and good‐tasting fruit with a short neck. The two lines are available upon request. A cross was made between YN and Jin5‐508 to create F_1_, which was self‐pollinated to generate the F_2_ progeny, and backcrossed with YN to generate for B_1_ or with Jin5‐508 for B_2_. The seedlings of Jin5‐508, YN, their F_1_ and F_2_ progeny and all 158 cucumber accessions (details in Table S5) were planted in the research greenhouse at Yangzhou University (Yangzhou, China). To allow full development of the cucumber fruit, only one well‐developed fruit among 5–10 nodes of a plant was retained. FNLs were phenotyped at 30 dpp. Cucumber fruits were cut lengthwise, and the FNL was recorded as the distance from the distal end of the pedicel to the endocarp. Data were collected from the mean values of six independent measurements from one fruit, because the fruit neck was not always straight.

### Scanning electron microscopy (SEM) imaging

For SEM, fruit necks were collected at 15 dpp, cut into 4 × 4‐mm pieces and fixed with 4% glutaraldehyde and stored at 4°C until use. The specimens were specific mounted on SEM stubs, sputter‐coated with gold–palladium and observed on a S‐4800 field emission SEM (Hitachi, Ibaraki, Tokyo, Japan) at an accelerating voltage of 10 kV.

The cell sizes of parental lines, D8 (wild type, WT) and transgenic fruits were estimated using SEM images with Image J software (https://imagej.nih.gov/ij/). The numbers of cells were counted using the cell counter plugin (http://rsbweb.nih.gov/ij/plugins/cell-counter.html) in Image J.

The size of the fields of counted cells was used to determine mean longitudinal sectional area per cell and, in combination with whole neck size, to calculate total cell number.

### QTL analysis using QTL‐seq

Two DNA pools (long‐necked pool and short‐necked pool) were constructed by mixing equal amounts of DNA from 50 long‐necked (FNL > 7.5 cm) and 50 short‐necked (FNL < 2.5 cm) F_2_ plants from the Autumn 2014 experiment. Total genomic DNAs from healthy leaves of Jin5‐508, YN and two extreme bulks were extracted using the CTAB method (Murray and Thompson, 1980). Equal amounts (5 μg) of genomic DNA were then used for paired‐end sequencing library construction. The four libraries were subjected to whole‐genome sequencing on an Illumina HiSeq2500 sequencer at Beijing Biomarker Technologies Corporation. After filtering, the high‐quality reads were mapped onto the 9930 cucumber reference genome (V2.0) (http://cucurbitgenomics.org/) using the Burrows‐Wheeler‐Alignment (BWA) tool and Genome Analysis Toolkit (GATK4.0.2.0) (Langmead and Salzberg, 2012; Li and Durbin, 2009). Those SNPs with read depth higher than 5 and base quality value higher than 20 were retained for QTL analysis. The well‐documented SNP index method was applied to calculate genotype frequency between the Sn‐bulk and Ln‐bulk that was satisfied by Δ (SNP_index) (Abe *et al.*, 2012; Takagi *et al.*, 2013). The SNP index at each SNP position was calculated as follows: SNP_index (Ln) = P_Ln_/(P_Ln_ + M_Ln_), SNP_index (Sn) = M_Sn_/(P_Sn_ + M_Sn_), and Δ(SNP_index) = SNP_index(Ln)−SNP_index(Sn). Here, P stands for Jin5‐508, M stands for YN, Ln denotes the genotype frequency from the long‐necked pool, and Sn denotes the genotype frequency from the short‐necked pool. If the pooled DNA comprises only the Jin5‐508 genome, then Δ(SNP index) = 1; if it is from the YN genome only, then Δ(SNP index) = ‒1; if both parents have the same SNP_index at the SNP position, then Δ(SNP index) = 0. It is expected that the larger the relative abundance, the higher is the possibility that the marker is associated with FNL. Only the QTL region with the loess‐fitted values of the markers above the threshold of the 99% of confidence interval was considered.

### Genetic map construction and QTL mapping

For genetic map construction, a single F_1_ plant from the cross between Jin5‐508 and YN was self‐pollinated to generate 135 F_2_ plants, from which 102 F_2:3_ families were generated for FNL data collection. FNL data were collected from at least 10 plants per family, and the means were used in the QTL analysis. Using the resequencing data, 40 SNPs and 40 InDels between the Jin5‐508 and YN were selected to construct a genetic map. Those SNPs and InDels fulfilled the following stringent criteria were used: (i) mapping quality filter equivalent to PASS; (ii) minimum read depth of 30; (iii) average base quality of the SNP ≥ 30; (iv) variant frequency ≥ 90%; and (v) on Chr7. For SNP genotyping, dCAPS markers were designed with the web‐based dCAPS Finder 2.0 (http://helix.wustl.edu/dcaps/dcaps.html). For InDels genotyping, only those with ≥5 bp differences were used for primer design with the web‐based Primer3 (v. 0.4.0, http://bioinfo.ut.ee/primer3-0.4.0/). A linkage analysis was carried out using JoinMap 4.0 software with the threshold LOD score of 2.5 (Kosambi, 1944). A QTL analysis was performed using the R/QTL package (http://www.rqtl.org/) with the composite interval mapping model (Broman *et al.*, 2003).

### Fine mapping and characterization of the *Fnl7.1* candidate gene

To narrow down the position of *Fnl7.1*, the flanking markers (SNP01 and InDel01) were used to genotype a large F_2_ segregating population to identify recombinants. The recombinants were then self‐pollinated to generate F_2:3_ families in which 15–20 plants per family were phenotyped. New SNP and InDel markers within the QTL region were developed, and genotype‐based haplotypes were constructed for the recombinants. Their relationships with the FNL phenotypes were evaluated to infer the most probable genomic region harbouring the *Fnl7.1* locus.

Gene prediction and functional annotation in the 14.1‐kb genomic DNA region was performed using the version 2 of Gy14 genome annotation (http://cucurbitgenomics.org/organism/16). We cloned the 14.1‐kb DNA sequences of the *CsFnl7.1* from Jin5‐508 and YN. Oligo synthesis and Sanger sequencing were conducted by Sangon Inc. (http://www.sangon.com/). To examine the expression dynamics of the candidate gene and the possible interactors, young fruits from Jin5‐508 and YN at 0, 3, 6, 9, 12 and 15 dpp were harvested and fruit necks were retained for total RNA extraction and first‐strand cDNA synthesis. qPCR was performed in triplicate on an iQ^TM^ 5 multicolour Real‐Time PCR detection system (Bio‐Rad, Hercules, CA, USA) using a RealMasterMix (SYBR Green) kit (Tiangen, Beijing, China). The specificity of the PCR amplification was verified by melt‐curve analysis (the gene‐specific primers are provided in Table S3). The relative expression level was calculated using the 2^−ΔΔCt^ method (Livak and Schmittgen, 2001) with cucumber *β‐actin* (GenBank AB010922) as an internal control.

### Expression of the *Fnl7.1* candidate gene in cucumber

The *CsFnl7.1* coding sequencing was amplified from Jin5‐508. After confirmation by Sanger sequencing, the cloned fragments were subsequently inserted into the pCAMBIA1301 vector carrying the CaMV 35S promoter to construct the 35S::CsFnl7.1 recombinant plasmid. Agrobacterium‐mediated cucumber transformation was carried out by a commercial service. Briefly, the 35S::CsFnl7.1 recombinant plasmid was delivered into *Agrobacterium tumefaciens* strain EHA105, which was transformed into the short‐necked cucumber line D8 (a North American type) using cotyledons as explants. Murashige and Skoog medium containing 50 mg/mL kanamycin was used to select transformants. The putative transgenic events were confirmed by PCR. T_2_ homozygous plants were used for phenotypic observations. Southern blot hybridization was performed to detect the transgene copy numbers following Xu *et al. *(2018).

### GUS assay constructs and histochemical staining

The 2.0‐kb promoter fragments immediately upstream of the ATG start codons were independently amplified by PCR using Jin5‐508 and YN genomic DNA as the templates. The fragments were then independently inserted upstream of the *GUS* gene in the binary vector PCAMBIA1301 using *Pst*I and *Nco*I. Fully expanded 5‐week‐old tobacco (*Nicotiana benthamiana*) leaves were infiltrated with *Agrobacterium tumefaciens* (OD600 = 0.5) harbouring the recombinant plasmids. The GUS activities of inoculated plants were measured by fluorometric quantitation of 4‐methylumbelliferone produced from the 4‐methylumbelliferyl β‐D‐glucuronide using a microplate reader (varioskan Flash, Thermo, Valtham, MA, USA).

### Y2H library screening and confirmation

We used the Matchmaker™ Gold Y2H library screening system (Clontech, Dalian, China) to screen the CsFnl7.1 interacting proteins. A normalized library was constructed by using equal amounts of cDNA obtained from necks of Jin5‐508 harvested at 0 and 3 dpp. The pGBKT7‐CsFnl7.1 construct was confirmed by Sanger sequencing, auto‐activation and toxicity test and then transformed into prey yeast strain Y2HGold. Screening of mated yeast cells was performed on starvation medium (SD) (–Leu/–Trp) and incubated at 28°C for 3 days. Potential interacting clones were further tested on a higher stringency SD/–Ade/–His/–Leu/–Trp media. The GED coding sequences of CsDRP6 and full‐length coding sequences of CsGLP1 were cloned separately into pGADT7 vector and cotransformed with pGBKT7‐CsFnl7.1, to verify their interaction. The Y2HGold strain containing pGBKT7‐53 and pGADT7‐RecT as a positive control, and pGBKT7‐Lam and pGADT7‐RecT as a negative control.

### Protein purification and GST pull‐down assay

The full‐length coding sequence of CsFnl7.1 was cloned to the pGEX‐6p‐1vector. The artificially synthesized GED coding sequences of CsDRP6 (containing start codon) and full‐length coding sequences of CsGLP1 were cloned separately into pet‐sumo vector. The recombinant plasmids were expressed in *Escherichia coli* strain Rosetta2 and induced by IPTG at 30°C for 3 h. Purification of the recombinant proteins was performed using BeyoGold™ GST‐tag Purification Resin and BeyoGold™ His‐tag Purification Resin (Beyotime Biotech, Shanghai, China). The pull‐down assay was performed by a commercial service (http://www.genecreate.com/). In brief, 100 μg of purified GST‐CsFNL7.1 protein and GST proteins were incubated with 200 μL 50% glutathione‐agarose beads for 60 min at 4°C. After centrifugation at 2500 *g* for 3 min at 4°C, the resulting beads were washed three times with 500 μL PBST buffer. Then, 100 μg of purified HIS‐CsDRP6 or HIS‐GLP1 was added to the Sepharose solution and incubated at 4°C overnight on a horizontal rotator. The beads were then washed three times with 1 mL PBST, and boiled in 100 μL bacterial lysates containing 50 μL 2 × SDS loading buffer and 50 μL RIPA Buffer for 10 min. Proteins were resolved by 12% SDS‐PAGE for Western blotting with anti‐GST and anti‐His antibodies.

### Geographical distribution and evolutionary analysis

The DIVA‐GIS7.5 software (http://swww.diva-gis.org/) was used to construct the geographical map. The variations (SNPs and InDels) in the *CsFnl7.1* promoter regions across 158 re‐sequenced lines were used to construct a phylogenetic tree. Whole‐genome resequencing reads of 17 lines were obtained from the National Center for Biotechnology Information (NCBI) database (Qi *et al.*, 2013); the remaining lines were re‐sequenced with Illumina HiSeq X Ten sequencer at Zhejiang Annoroad Biotechnology Co., Ltd. (Yiwu, Zhejiang, China). Raw reads were filtered with the FASTX Toolkit (http://hannonlab.cshl.edu/fastx_toolkit/), and those reads with adapters or quality scores ≤ 30 (fastq_quality_trimmer and fastq_quality_filter) were removed. The remaining clean reads were mapped against 9930 V2.0 reference genome to call SNPs and InDels within the *CsFnl7.1* promoter region by using the BWA‐GATK workflow. SNPs and InDels were filtered based on default parameters with some modifications (read depth ≥ 15, quality score ≥ 30, variant frequency ≥ 90%). Multiple sequences alignment and neighbour‐joining tree construction were performed using MEGA‐X software (Kumar *et al.*, 2018) with 1000 bootstrap replications.

## Results

### Phenotypic characterization and inheritance of FNL

**Figure 1:**
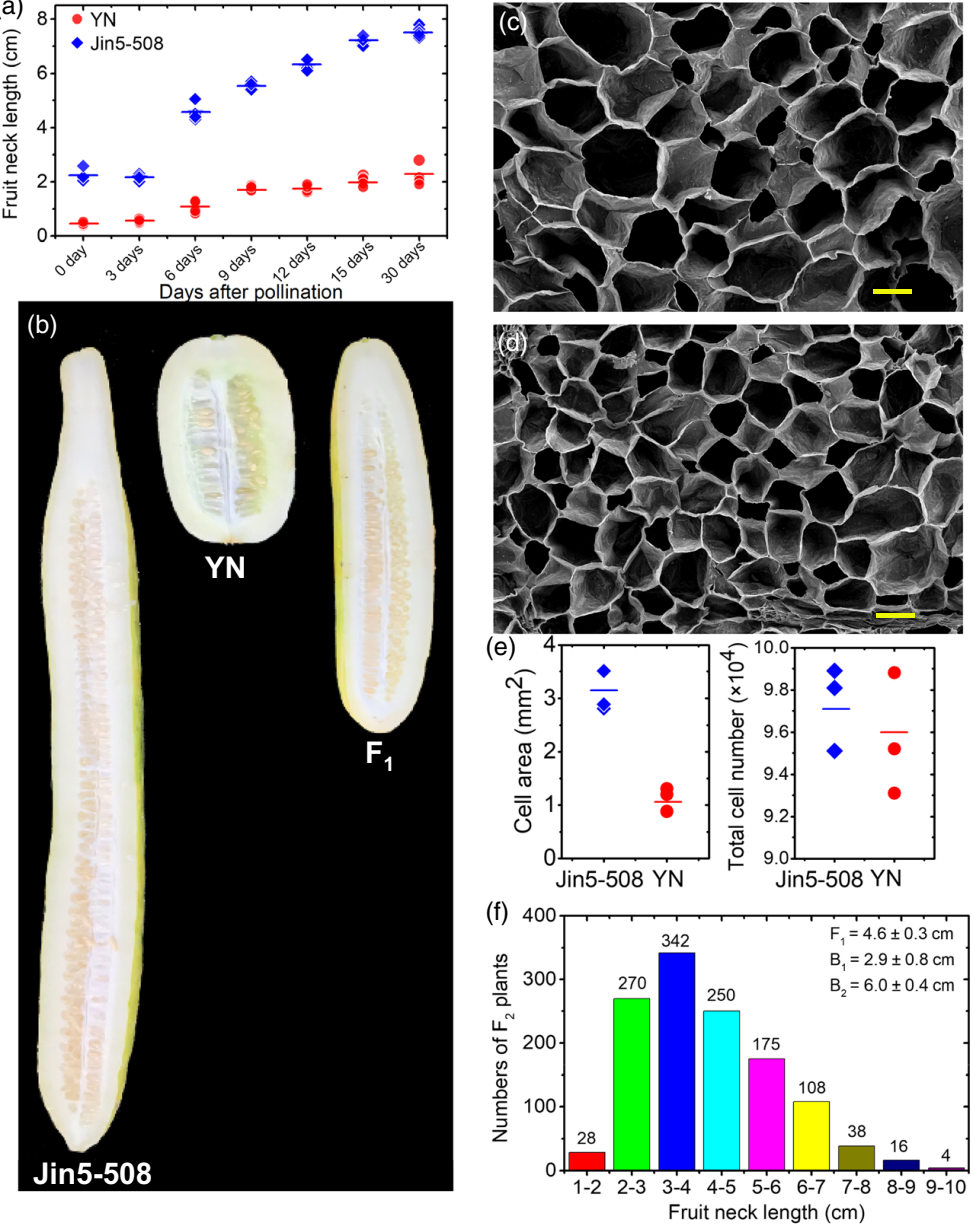
Phenotypic differences in cucumber fruit neck length (FNL). (a) Changes in FNL during the 30‐days fruit developmental period in Jin5‐508 and YN. (b) FNL variations in Jin5‐508, YN and their F_1_ progeny. Scanning electron microscopy of the longitudinal sections of the fruit necks of Jin5‐508 (c) and YN (d) harvested at 15 days post‐pollination. Bar = 100 μm. (e) Comparison of the average cell area (*n* = 100) and total cell number between the necks of Jin5‐508 and YN harvested at 15 days after self‐pollination. The short lines show the means of three biological replicates. Each value denotes the mean of 10–15 non‐overlapping fields of view. (f) Frequency distribution of the FNL measurements among 1231 F_2_ individuals in spring 2014. F1:Jin5‐508 × YN; B_1_: F_1_ × YN; B_2_: F_1_ × Jin5‐508.

Because there were no significant increases in the neck length in either Jin5‐508 or YN at 30 d post‐pollination (dpp), FNL was measured at this stage (Figure 1a). Images of typical mature fruits for the two parental lines and their F_1_ are shown in Figure 1b. The mean FNLs for the long‐necked parent Jin5‐508 and short‐necked parent YN were 7.5 cm and 2.2 cm, respectively. The cell areas (*n* = 100) in necks of Jin5‐508 (Figure 1c) were significantly greater than those in YN (Figure 1d), whereas the total cell numbers does not significantly differ between the two lines (Figure 1e), suggesting that the long neck phenotype of Jin5‐508 might be to the result of an increase in cell expansion. The mean FNL for the Jin5‐508 × YN F_1_ hybrid was 4.6 cm, which is close to the midpoint between the parental means. The FNL distributions in both backcross families (B_1_ and B_2_) shifted towards that of the recurrent parent (Figure 1f). The FNL frequency distributions among 1231 F_2_ segregating plants (Spring 2014) showed continuous variation and were close to a normal distribution curve, suggesting that FNL is quantitatively inherited.

### Identification of a major‐effect QTL for FNL through QTL‐seq

**Figure 2:**
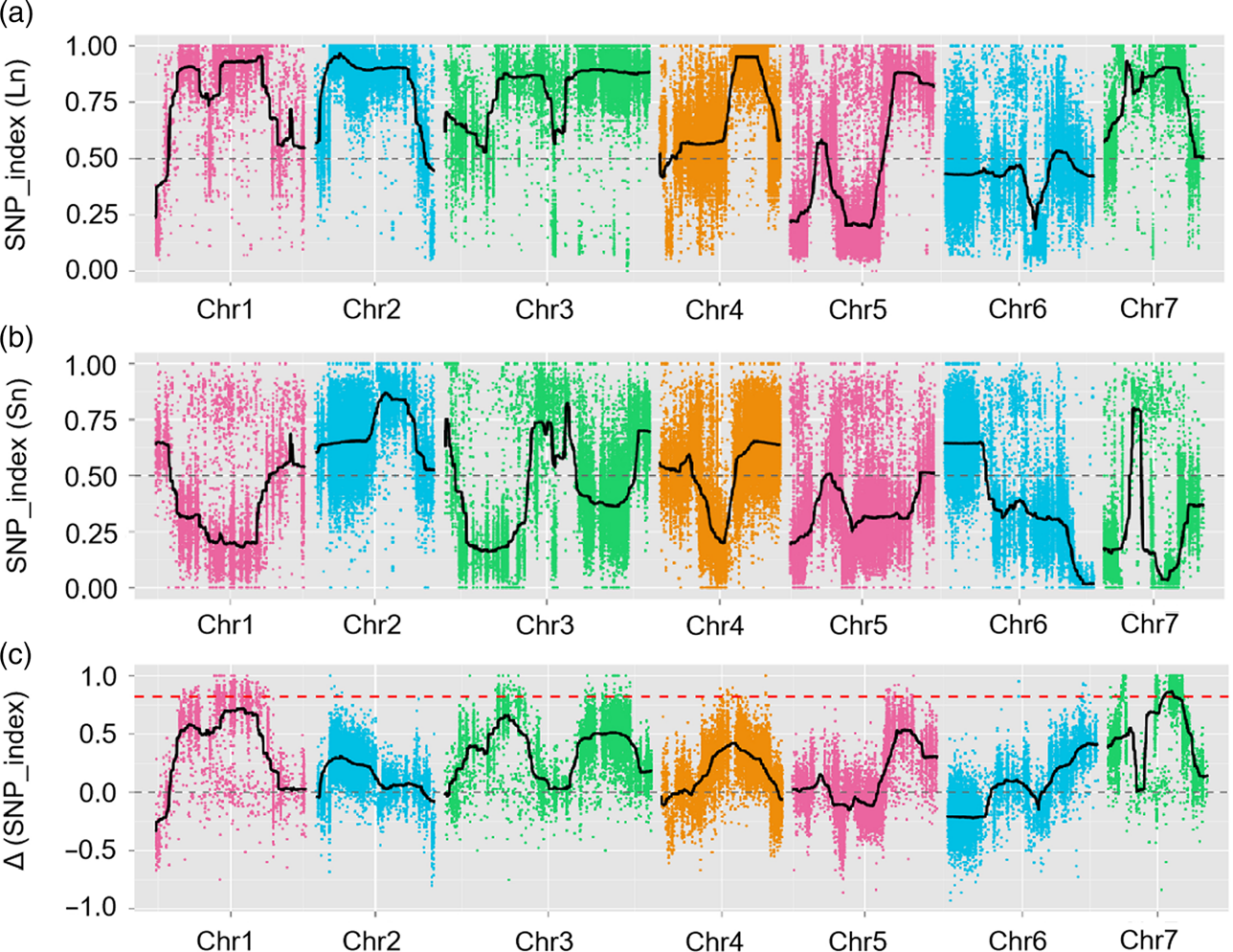
QTL‐seq approach for mapping genomic regions controlling cucumber fruit neck length. (a) SNP_index plot for the long‐necked (Ln) pool; (b) SNP_index plot for the short‐necked (Sn) pool; (c) ∆SNP_index plot. ∆SNP_index = SNP_index (Ln) − SNP_index (Sn). The greater the result of Δ (SNP_index), the stronger the association with the phenotype. The red dotted line represents the threshold (0.833) of Δ (SNP_index), which was calculated by Loess regression. Chr, chromosome; SNP, single nucleotide polymorphism.

Among the 1231 F_2_, 50 long‐necked (Ln) and 50 short‐necked (Sn) individuals were selected, and two extreme DNA pools were created. Illumina high‐throughput sequencing generated 97.78 million and 82.16 million pair‐end reads (150 bp in length) in the Ln and Sn pools, respectively. A total of 55.04 million and 54.89 million paired‐end reads were obtained for Jin5‐508 and YN, respectively. The average sequencing depths were 34‐fold in Jin5‐508, 20‐fold in YN, 61‐fold in the Ln pool and 52‐fold in the Sn pool (Table S1). On average, 90.9% of the reads from both parents and the bulked samples were mapped to the cucumber 9930 genome (v2.0). In total, 342 496 single nucleotide polymorphisms (SNPs) in the Ln pool and 399 764 SNPs in the Sn pool were identified after their alignments with the reference genome. The Δ (SNP index) was calculated by integrating the SNP index values (Table S2) of the Ln pool (Figure 2a) and Sn pool (Figure 2b) and plotting them against the 9930 genome with a statistical confidence interval (threshold = 0.833). The calculation revealed the presence of a major‐effect QTL controlling FNL at the 9.98–14.61 Mb (above the red dotted line) on chromosome 7 (Chr7), which was designated as *Fnl7.1* (Figure 2c). The QTL *Fnl7.1* region displayed an average SNP index higher than 0.75, with the highest SNP index value being 0.87 in Ln‐bulk (Figure 2a); values were lower than 0.25 in Sn‐bulk, with the lowest value being 0.03 (Figure 2b).

### Validation of the major‐effect QTL using traditional QTL mapping

**Figure 3:**
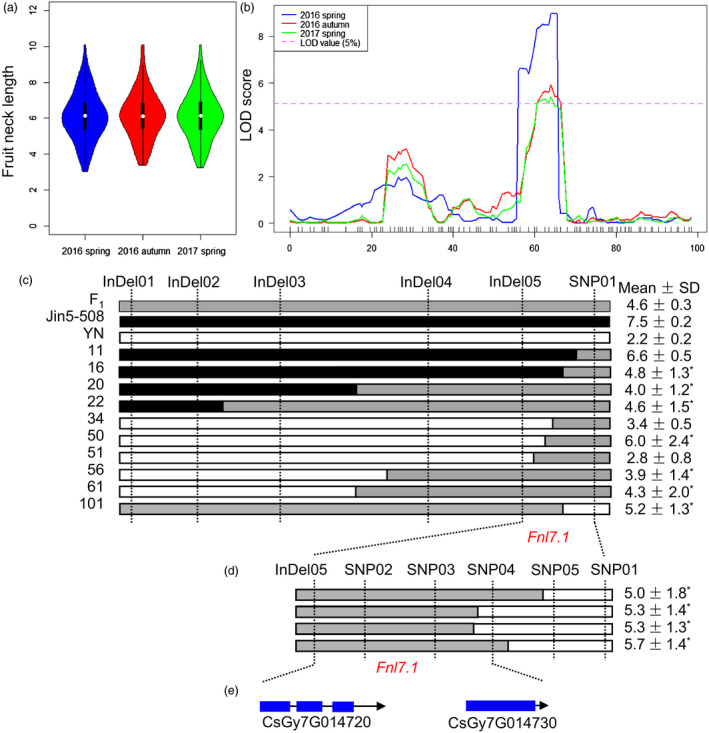
Mapping of the cucumber *Fnl7.1* locus. (a) Violin plot depicting the phenotypic distributions of cucumber fruit neck lengths among 102 Jin5‐508 × YN F_2:3_ in three environments (Spring 2016, Autumn 2016 and Spring 2017). (b) Logarithm of the odds (LOD) profiles of fruit neck length quantitative trait loci (QTL) detected on the local genetic linkage map of cucumber chromosome 7 in three environments (Spring 2016, Autumn 2016 and Spring 2017). The x‐axis is map genetic position (cM). (c) Genotyping of recombinant plants from 4000 F_2_ individuals of the Jin5‐508 × YN cross. (d) Genotyping of recombinant plants from additional 7600 F_2_ individuals of the Jin5‐508 × YN cross. The fruit neck lengths (FNLs) of the two parents, their F_1_ and each recombinant are shown on the right. Open, grey and filled bars represent homozygous fragments from YN, possible crossover intervals and homozygous fragments from Jin5‐508, respectively. The recombinants were identified, and each was self‐pollinated to produce F_2:3_ families. Mean (±SDs, from 10 to 15 F_2:3_ individuals) FNLs of each haplotype are shown on the right. *Segregated in the F_2:3_ family. (e) Annotation of the 14.1‐kb region. Blue boxes indicate exons, and black lines indicate introns. Arrow indicates the orientation of the gene.

To verify the accuracy of the major‐effect QTL‐governing FNL identified by QTL‐seq, classical QTL mapping was performed. Phenotypic data for the 102 Jin5‐508 × YN F_2_‐derived F_2:3_ families were obtained in three environments (Spring 2016, Autumn 2016 and Spring 2017). The observed distribution of the F_2:3_ family also followed a largely normal distribution and covered a large length range in each environment (Figure 3a). Using the biparental resequencing data, 80 markers (40 SNPs and 40 InDels) on Chr7 were screened, of which 72 were polymorphic between Jin5‐508 and YN, and applied to the 102 F_2_ mapping individuals. The resulting genetic map had 66 marker loci in one linkage group spanning 97.8 cM and physically covering 19.2 Mbp or 99% of Chr7. Using the composite interval mapping method and the phenotypic data for FNL among the 102 F_2:3_ families, only one major‐effect QTL was identified successfully in all three environments. The phenotypic variations explained by the QTL (*R^2^*) ranged from 27.9% to 32.7%, with peak logarithm of the odds (LOD) values ranging from 5.4 to 9.0 (Figure 3b). The LOD profiles for the QTL from the three environments completely overlapped, with the same 2.0 LOD‐support interval (11.75–15.66 Mb) and peak marker. This conventional biparental QTL mapping result was consistent with the QTL‐seq and supported the presence of a major QTL for FNL, *Fnl7.1*, at an overlapping genomic interval of approximately 2.85 Mb (11.75–14.6 Mb) on Chr7.

### Fine mapping delimited the *Fnl7.1* locus into a 14.1‐kb region containing two predicted genes

To narrow down the position of *Fnl7.1*, 4,000 F_2_ plants (including 1231 plants for bulk construction) were genotyped with InDel01 and SNP01, and 51 recombinant plants were obtained. Four new InDel markers (InDel02–InDel05) were developed (see Table S3 for primer information) and used to genotype the 51 recombinants. Genotypic data of the 6 markers and 10 representative haplotypes are illustrated in Figure 3c. The mean FNLs of the 10 haplotypes are shown in Figure 3c (on the right side), 3 and 4 of which were long and short, respectively. Thus, the *Fnl7.1* locus must reside in the 25.4‐kb region defined by InDel05 (14 367 083 bp) and SNP01 (14 392 514 bp). For a more precise QTL location, we screened an additional 7600 Jin5‐508 × YN F_2_ plants with the two flanking markers InDel05 and SNP01. Four new recombinants were identified, and each was self‐pollinated to produce F_2:3_ families. The two recombinants were genotyped with additional four SNP markers (SNP02‐SNP05). Inspection of the genotypic and phenotypic data from F_2:3_ placed *Fnl7.1* in the 14.1‐kb region defined by InDel05 and SNP04 (Figure 3d). In the 14.1‐kb region, only two genes were predicted (Figure 3e). *CsGy7G014720* was predicted to encode a protein belonging to the late embryogenesis abundant (LEA) family. *CsGy7G014730* was predicted to encode a transcription repressor containing the OVATE domain.

### *CsFnl7.1* was the candidate gene for *Fnl7.1*

We cloned and sequenced the 14.1‐kb genomic DNA sequences from Jin5‐508 and YN. Alignment of the sequences identified 17 SNPs or InDels within this region, of which three were located in the intergenic region, one silent mutation in the first intron of *CsGy7G014720*, three synonymous mutations in the exons of *Gy7G014720*, and the remaining were occurring in the promoter region of *CsGy7G014720* (including the InDel05, Figure S1)*.* Furthermore, the marker InDel05 was co‐segregating with *Fnl7.1* within the 25.4‐kb interval (Figure 3c). These evidence suggested that *CsGy7G014720* (designated as *CsFnl7.1* hereinafter) is the possible candidate gene for the *Fnl7.1* locus.

**Figure 4:**
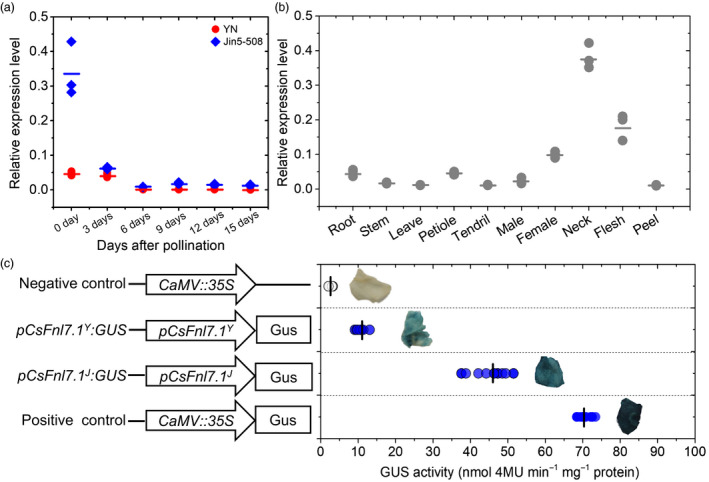
Expression analyses. (a) Quantitative PCR expression analysis of *CsFnl7.1* in necks of Jin5‐508 and YN. (b) Quantitative PCR expression analysis of *CsFnl7.1* in different tissues of Jin5‐508. Each circle denotes the mean relative expression level of three replicates. The short lines show the means of three biological replicates. (c) Quantitative and qualitative analyses of the activity of the *CsFnl7.1* promoter in tobacco leaves based on GUS histochemical staining. *pCsFnl7.1^Y^*: *CsFnl7.1* promoter cloned from YN; *pCsFnl7.1^J^*: CsFnl7.1 promoter cloned from Jin5‐508. The circles are given as the means of three independent measurements. The short lines show the means of ten biological replicates.

We proposed that the gene underlying the *Fnl7.1* locus might be differentially expressed, resulting in variation in FNL. Thus, we examined the expression dynamics of *CsFnl7.1* in Jin5‐508 and YN at 0, 3, 6, 9, 12 and 15 dpp using qRT‐PCR. As expected, *CsFnl7.1* exhibited consistent patterns in the long‐necked Jin5‐508 and the short‐necked YN. The expression levels of *CsFnl7.1* in the necks of both lines continued to decrease after pollination at all six time points, but they were significantly higher in Jin508‐28 than in YN at 0 day after pollination (Figure 4a). A tissue‐specific expression analysis revealed that *CsFnl7.1* was expressed in all 10 tested tissues (root, stem, leave, petiole, tendril, male flowers, female flowers, neck, flesh and peel), with the highest expression occurring in neck (Figure 4b). These data further suggested that *CsFnl7.1* is a good candidate for the *Fnl7.1* locus.

### A *CsFnl7.1* promoter polymorphism is associated with *CsFnl7.1* expression

Promoters are the genetic elements associated with cis‐regulation of gene expression (Riewe *et al.*, 2016). To investigate the causes of the observed differences in *CsFnl7.1* allelic expressions, we focused on polymorphisms in the gene’s promoter region. We cloned the promoter sequence of the Jin5‐508 (*pCsFnl7.1^J^*) and YN (*pCsFnl7.1^Y^*) alleles and detected their activities using an Agrobacterium‐mediated GUS transient assay in tobacco leaves. Sequences alignment between the two lines revealed the existence of 25 SNPs and 7 InDels in the promoter region (Figure S2). Both promoter constructs led to significant increases in GUS activity levels relative to the vector control alone (negative control), with an approximately fourfold greater increase for *pCsLEA^J^* than for *pCsLEA^Y^* (Figure 4c). This result is in good agreement with the higher *CsFnl7.1* mRNA expression levels in Jin5‐508 than in YN, and it supports the assumption that *CsFnl7.1* expression is cis‐regulated.

### Transgenic *CsFnl7.1* cucumber plants exhibit greater FNLs

**Figure 5:**
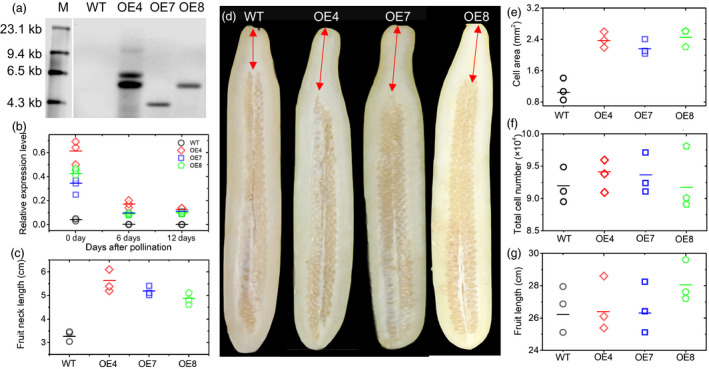
Cucumber transgenic analysis. (a) Southern hybridization results of wild type (WT) and T_2_ transgenic overexpression lines OE4, OE7 and OE8. The gDNA was digested with *Kpn*I and hybridized with a digoxigenin‐labelled NPT II probe. M indicates the marker. (b) Confirmation of *CsFnl7.1* expression levels in the WT and transgenic cucumber lines as assessed by quantitative PCR. Each value denotes the mean relative expression level of three replicates. (c) Comparison of average fruit neck lengths of WT and transgenic lines at 40 days after self‐pollination. Each value denotes the mean of 3–10 fruits. (d) Phenotypes of WT and three independent transgenic cucumber lines (OE4, OE7 and OE8) at 40 days after self‐pollination. (e) The average cell areas (*n* = 100) in the necks of WT and transgenic lines harvested at 40 days after self‐pollination. Each value denotes the mean relative expression level of three replicates. (f) Comparison of total cell numbers of WT and transgenic lines at 40 days after self‐pollination. Each value denotes the mean of 10–15 non‐overlapping fields of view. (g) Comparison of average fruit lengths of WT and transgenic lines at 40 days after self‐pollination. Each value denotes the mean of 3–10 fruits. The short lines show the means of three biological replicates.

To determine whether *CsFnl7.1* controls cucumber FNL, we generated transgenic plants overexpressing *CsFnl7.1* in the short‐necked cucumber ‘D8’. Three T_2_ transgenic lines—OE4, OE7 and OE8—were obtained and validated by Southern hybridization to obtain the numbers of fragments and by qRT‐PCR to investigate the transcript abundance levels. No hybridizing band was observed in the wild type (WT), whereas OE4, OE7 and OE8 showed two, one and one band, respectively, suggesting that OE4 contained two inserts, while OE7 and OE8 each contained one insert (Figure 5a). In comparison with the WT, the three transgenic lines displayed significantly higher expression levels (Figure 5b) and longer FNLs (Figure 5c,d) than the WT, suggesting that CsFnl7.1 does function to affect FNL in cucumber. The average cell areas (*n* = 100) in the necks of OE4, OE7 and OE8 were 2.45 ± 0.42, 2.29 ± 0.16 and 2.57 ± 0.11 mm^2^, respectively, while in WT, the area was 1.04 ± 0.23 mm^2^ (Figure 5e). However, no significant changes were observed in the total cell numbers (Figure 5f) and lengths (Figure 5g) of mature fruits between the OE lines and WT, further confirmed that CsFnl7.1 might regulate neck length by modulating cell expansion.

### CsFnl7.1 interact with cell expansion related proteins

To identify the potential proteins interactors of CsFnl7.1, we conducted a yeast two‐hybrid (Y2H) screen using CsFnl7.1 protein as the bait and a cDNA library from necks of Jin5‐508 harvested at 0 and 3 dpp as the prey. We screened 2 × 10^6^ yeast transformants and identified 53 positive clones (Table S4). Of the particular interest were several proteins that involving in cell expansion (see discussion), including Csa5G647440 (dynamin‐related protein 6, CsDRP6), Csa7G450510 (germin‐like protein 1, CsGLP1), Csa4G025060 (ras‐related protein 1A), Csa4G000580 (α‐tubulin) and Csa7G027790 (cysteine proteinase 1).

**Figure 6:**
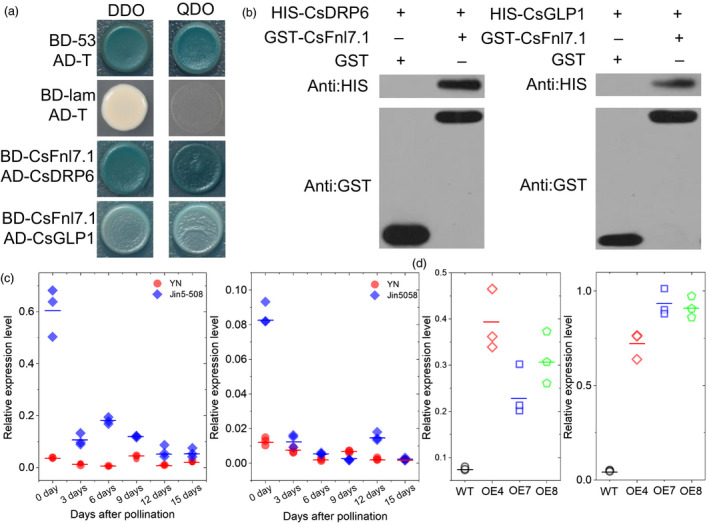
CsFnl7.1 can interact with CsDLP6 and CsGLP1. (a) Yeast two‐hybrid analysis. CsFnl7.1 protein was fused to the DNA‐binding domain (BD) to generate the prey construct. CsDLP6 and CsGLP1 proteins were fused to the GAL4 activation domain (AD) to generate the bait constructs. DDO, double drop‐out medium (SD/‐Trp‐Leu) containing X‐α‐gal; QDO, quadruple drop‐out medium (SD/‐Trp‐Leu‐His‐Ade) containing X‐α‐gal. BD‐lam is a negative control that encodes a fusion of human lamin C protein, and the GAL4 DNA‐BD. BD‐53 and AD‐T are positive control that encode a fusion of murine p53 protein and the GAL4 DNA‐BD or a fusion of SV40 large T antigen and the GAL4 DNA‐AD. (b) *In vitro* pull‐down analysis using anti‐HIS and anti‐GST antibodies. The left lanes are control groups using GST and HIS‐CsDLP6 or HIS‐CsGLP1 as inputs. The right lanes are test groups using GST‐CsFnl7.1 and HIS‐CsDLP6 or HIS‐CsGLP1 as inputs. Using anti‐HIS Abs, the presence of a band indicates that the pulled down proteins contain HIS‐CsDLP6 or HIS‐CsGLP1 in the test group, suggesting that the HIS‐fusioned proteins could be pulled down by GST‐CsFnl7.1 but not by GST only. Using anti‐GST Abs, the presence of bands further verified the interactions. (c) Quantitative PCR expression analysis of *CsDRP6* and *CsGLP1* in necks of Jin5‐508 and YN. (d) Confirmation of *CsDRP6* and *CsGLP1* expression levels in the necks of transgenic cucumber lines at 0 day post‐pollination as assessed by quantitative PCR. WT: wild type (D8). OE4, OE7 and OE8 are the three CsFnl7.1 overexpression lines. Each value denotes the mean relative expression level of three replicates. The short lines show the means of three biological replicates.

To verify the protein interactions, the dynamin GTPase effector domain (GED, 747‐828 aa) coding sequences of CsDRP6 and full‐length coding sequences of CsGLP1 were cloned separately into pGADT7, and the full‐length coding sequences of CsFnl7.1 were cloned into pGBKT7 (BD). Yeast strains cotransformed with CsGLP1‐AD or GED‐AD and CsFnl7.1‐BD grew normally on double drop‐out medium (SD/‐Trp‐Leu) and quadruple drop‐out medium (SD/‐Trp‐Leu‐His‐Ade) containing X‐a‐Gal (Figure 6a). In order to further confirm the interactions, an *in vitro* GST pull‐down assay was performed. We generated three recombinant proteins, GST‐CsFnl7.1, HIS‐CsDRP6 and HIS‐CsGLP1. The fusion protein HIS‐CsDRP6 and HIS‐CsGLP1 was effectively captured by GST‐CsFnl7.1, whereas was not captured by GST (Figure 6b). The result further confirmed that CsFnl7.1 physically interact with CsDRP6 and CsGLP1. Furthermore, we found the expression dynamics of *CsDRP6* and *CsGLP1* in Jin5‐508 and YN at 0, 3, 6, 9, 12 and 15 dpp (Figure 6c) were similar to those in CsFnl7.1 (Figure 4a). Notably, the expressions of *CsDRP6* and *CsGLP1* were up‐regulated in the fruit necks of CsFnl7.1 overexpression lines (Figure 6d), suggesting that the three genes may function in the same pathway in regulating FNL in cucumber.

### Geographical distribution and evolution analysis of *Fnl7.1* in a natural population

**Figure 7:**
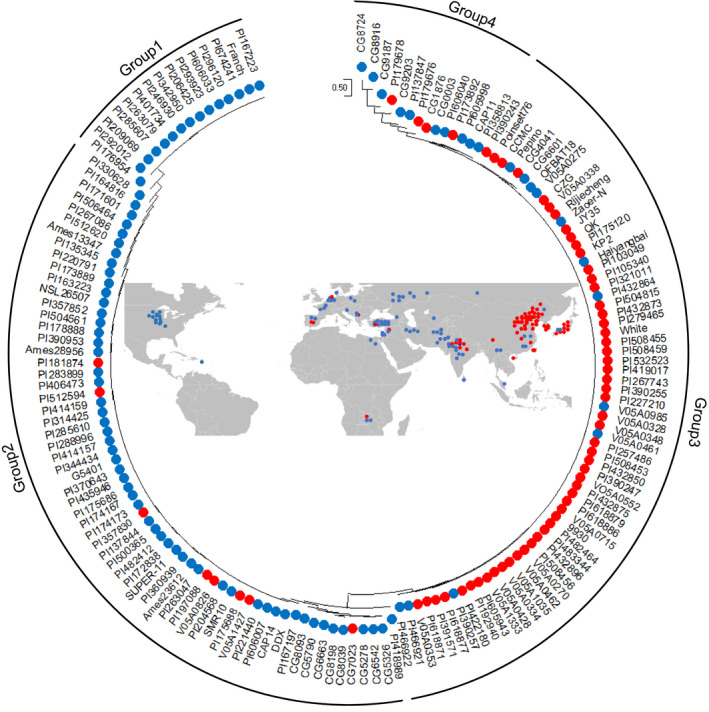
Phylogenetic analysis (outer) and geographical distribution (inside) of the *Fnl7.1* locus. The dendrogram was constructed using the SNPs and InDels within the promoter regions of *CsFNL7.1* across 158 cucumber accessions. To clarify the relationships, we defined those accessions with fruit neck lengths (FNLs) longer than 4.2 cm (mean FNL value of the 158 cucumber accessions) as long‐necked (red solid dots), while those shorter than 4.2 cm were defined as short‐necked (blue solid dots). Details of the phenotypes and genotypes are listed in Tables S5 and S6, respectively.

To determine phenotypic differences between the geographical regions, we plotted the approximate geographical distribution of the 158 cucumber accessions (see Table S5 for FNL phenotype) on a world map. Among the 158 accessions, 154 were cultivated cucumbers: 62 from East Asia, 71 from Eurasia and 21 from India. In addition, two each belonged to wild CSH and semi‐wild Xishuangbanna (XIS). To clarify the relationships, we defined those accessions with FNLs longer than 4.2 cm (mean FNL value of the 158 cucumber accessions) as long‐necked and those shorter than 4.2 cm as short‐necked. The accessions from East Asian were highly enriched with the long‐necked phenotype (53 out of 62), whereas 64 of 71 Eurasia accessions presented the short‐necked phenotype (Figure 7). Among the 21 India lines, 13 and 8 belonged to long‐ and short‐necked, respectively. The results suggested that the *Fnl7.1* locus might have originated from India. To confirm this and further investigate the distribution of *Fnl7.1* alleles in natural populations, a phylogenetic tree was constructed using variations in the *CsFnl7.1* promoter region across 158 re‐sequenced lines having FNL data. Genotypes of the 158 cucumber accessions at the *CsFnl7.1* promoter region are presented in Table S6. In the resulting neighbour‐joining tree, the 158 lines were divided into four groups, with groups 1 and 2 containing predominantly short‐necked accessions from Eurasia (62 out of 80), and group 3 containing mainly long‐necked accessions from East Asia (55 out of 72). For the 21 cultivated accessions from India, 8, 4, 7 and 3 were assigned to groups 1, 2, 3 and 4, respectively. In addition, the two XIS and two CSH from India were clustered into group 4 (Figure 7). These results further confirmed the hypothesis that the *Fnl7.1* alleles originated from India.

## Discussion

In the present study, a major‐effect QTL for FNL in cucumber, *Fnl7.1*, was successfully identified using bulked segregant analysis combined with NGS‐based whole‐genome resequencing to genotype genome‐wide SNPs in the long‐necked parent Jin5‐508 and the long‐necked progeny DNA pool, as well as in the short‐necked parent YN and the short‐necked progeny DNA pool. Subsequently, the SNP index was used to perform accurate, quantitative assessments of the frequencies of parental alleles, as well as the genomic contributions from the two parents to the F_2_ individuals. The method has proven to be cost‐effective and efficient for QTL identification in cucumber (Lu *et al.*, 2014; Win *et al.*, 2017; Xu *et al.*, 2018; Xu *et al.*, 2015), as well as in other plant species, such as chickpea (Singh *et al.*, 2016) and groundnut (Pandey *et al.*, 2017). The derived QTL, *Fnl7.1*, was validated by traditional QTL mapping, indicating the validity of the major‐effect QTL‐governing FNL in cucumber. The integration of QTL‐seq with traditional QTL mapping confirmed the location of *Fnl7.1* at an overlapping genomic interval of approximately 2.85 Mb (11.75–14.6 Mb) on Chr7. With additional recombinants and molecular markers, the *Fnl7.1* locus was finally reliably delimited into a 14.1‐kb region, in which two genes were predicted, including *CsFnl7.1*. The results of the fine mapping laid a solid foundation for revealing the causal gene regulating FNL in cucumber. The combined evidence from sequences alignment analysis (Figure S1), qPCR (Figure 4a, b) and the cucumber transgenic study (Figure 5) all support *CsFnl7.1* being the most likely candidate gene responsible for FNL variation. Our work illustrated how major‐effect QTL can quickly be cloned through the combined use of QTL‐seq and fine genetic mapping in segregated populations. GUS histochemical staining revealed that a *CsFnl7.1* promoter polymorphism is associated with *CsFnl7.1* expression (Figure 4c). An alignment of genomic DNA sequences between the parental lines identified 25 SNPs and 7 InDels in the promoter region of *CsFnl7.1* (Figure S2)*.* Previous studies have revealed strong associations between fruit length and neck length (Fanourakis and Tzifaki, 1992; Yuan *et al.*, 2008). Consistent with these observations, the correlation between the two traits was significant in the 1,231 F_2_ plants (Pearson's correlation coefficients *r* ^2^ = 0.68, *P* = 0.05) derived from Jin5‐508 and YN, suggesting that the two traits may share at least some common genetic factors. However, significant changes in fruit length between *CsFnl7.1* transgenic lines and WT were not observed (Figure 5g). Thus, we assumed that QTL *Fnl7.1* does not affect longitudinal fruit growth. In the present study, the QTL‐seq approach was applied to map the major‐effect QTL(s), which might explain why the co‐localized QTLs were not detected. In fact, we identified a major‐effect QTL on Chr1 (*fl1.1*) controlling the fruit length using the same F_2_ population (from same season but not the same extreme individuals) and mapping strategy (Liu, 2018). *fl1.1* correspond well to the fruit length QTL identified in Weng *et al. *(2015) and Pan *et al. *(2017).

LEA proteins were first reported as abundant in the later stages of cotton seed embryogenesis (Dure *et al.*, 1981) and were subsequently found to be expressed in vegetative and reproductive tissues (Hundertmark and Hincha, 2008). The *Arabidopsis* genome encodes 51 putative members of the LEA protein family, which can be divided into nine subfamilies (Hundertmark and Hincha, 2008). Despite the most prominent function of LEAs being to protect cells from damage caused by water limitation (Olvera‐Carrillo *et al.*, 2011), some LEAs are involved in controlling plant development. For example, *SAG21* (*AtLEA5*, *At4g02380*) encodes a mitochondria‐localized LEA protein; its antisense lines exhibit reduced primary root lengths, while overexpression lines show longer root hairs (Salleh *et al.*, 2012). The rice gene *HVA1*, which encodes a group 3 small LEA protein, promotes primary and lateral root elongation through the regulation of signalling and homeostasis of auxin and abscisic acid (Chen *et al.*, 2015). Here, the LEA‐encoding gene *CsFnl7.1* was responsible for increased FNLs in cucumber fruit, and this may represent a novel function for this protein family. Our morphological data showed that the cell sizes differed between the parental lines. Thus, we hypothesized that *CsFnl7.1* might regulate fruit neck development by modulating cell expansion. This hypothesis was supported by the protein–protein interaction studies. Directly interactions between CsFnl7.1 and CsDRP6 or CsGLP1 were confirmed *in vitro* by complementary Y2H assays and GST pull‐down experiments (Figure 6). Previous studies showed that GLPs play essential role in maintaining cell dimension in rice (Banerjee and Maiti, 2010), participates in cell wall expansion in cotton (Kim *et al.*, 2004) and cell growth in *Pinus caribaea* (Mathieu *et al.*, 2003) and mediates cell expansion in an auxin dependent manner in *Prunus salicina* (El‐Sharkawy *et al.*, 2010). Dynamin and DRPs are high‐molecular mass GTP binding proteins that are involved in membrane tubulation and vesiculation (Xiong *et al.*, 2010). Mutant phenotypes have provided important insight into the functions of DRPs. Kang *et al. *(2003) observed that *adl1A* (same to *drp1A*) and *adl1E* (same to *drp1E*) double mutations result in embryo lethal with disturbed cytokinesis and cell expansion. Collings *et al. *(2008) also observed that the widespread defects in endocytosis, cellulose synthesis, cytokinesis and cell expansion in the *Arabidopsis rsw9* (radial swelling) mutant were caused by a mutation in *DRP1A* (*At5g42080*).

The results of the phylogenetic analysis revealed the presence of four subgroups among the 158 investigated cucumber accessions (Figure 7). The results presented support the findings of Qi *et al. *(2013), in which natural cucumber populations could be roughly classified into East Asian, Eurasian, India and XIS groups. In general, the Eurasian accessions—mainly American pickle and European slicer cucumber varieties—have a short neck or no neck; while East Asian accessions—mainly north China and Japanese long cucumber varieties—exhibit the long neck phenotype (Fanourakis and Tzifaki, 1992). This is supported by our geographical distribution and phylogenetic analysis (Figure 7). We believe that the relatively common occurrence of the short neck alleles in Eurasian accessions and long‐necked alleles in East Asian accessions may be a consequence of human selection with delineated breeding efforts. Interestingly, we also found that five accessions from East Asian were assigned to group 1, and eight accessions from Eurasian were assigned to group 3. The exception was likely the result of cucumber germplasm exchange between the two geographical regions (Wang *et al.*, 2018). Despite the low numbers of accessions included, we found that the Indian accessions had relative higher genetic and phenotypic diversity levels. Because the cultivated cucumber is indigenous to India, it is reasonable to speculate that the *Fnl7.1* locus originated in India and underwent diversifying selection for specialized market classes during the use of Indian germplasm in cucumber breeding (Qi *et al.*, 2013). Additionally, the shorter neck length would increase the consumer appeal of the cucumber, encouraging consumption, which would promote seed dispersal.

## Conflict of interests

The authors declare that they have no competing interests.

## Author contributions

X.C., X.Q. and Q.X. conceived the experiment. X.X., C.W. and Q.L. performed the research. C.W., W.Q. and Q.L. collected the data. X.X. analysed the data and wrote the manuscript. All authors reviewed and approved this submission.

## Supporting information


**Figure S1** Alignment of the 14.1‐kb genomic DNA sequences from Jin5‐508 and YN.

**Figure S2** Alignment of the promoter sequences of Jin5‐508 and YN.

**Table S1** Summary of the sequencing data and mapping results.

**Table S2** Detailed information of SNP_index values for the long‐necked (Ln) and short‐necked (Sn) pools.

**Table S3** Information on the primers used in this study.

**Table S4** The potential proteins interactors of CsFnl7.1 from yeast two‐hybrid library screening.

**Table S5** Information on the 158 cucumber accessions used for the phylogenetic analyses.

**Table S6** Genotypes of SNPs and InDels within the promoter region of *CsFNL7.1* across the 158 cucumber accessions.

## References

[pbi13326-bib-0001] Abe, A. , Kosugi, S. , Yoshida, K. , Natsume, S. , Takagi, H. , Kanzaki, H. , Matsumura, H. *et al*.(2012) Genome sequencing reveals agronomically important loci in rice using MutMap. Nat. Biotechnol. 30, 174–178. 22267009 10.1038/nbt.2095

[pbi13326-bib-0002] Banerjee, J. and Maiti, M.K. (2010) Functional role of rice germin‐like protein1 in regulation of plant height and disease resistance. Biochem. Biophys. Res. Comm. 394, 178–183. 20188068 10.1016/j.bbrc.2010.02.142

[pbi13326-bib-0004] Broman, K.W. , Wu, H. , Sen, S. and Churchill, G.A. (2003) R/qtl: QTL mapping in experimental crosses. Bioinformatics 19, 889–890. 12724300 10.1093/bioinformatics/btg112

[pbi13326-bib-0005] Che, G. and Zhang, X. (2019) Molecular basis of cucumber fruit domestication. Curr. Opin. Plant Biol. 47, 38–46. 30253288 10.1016/j.pbi.2018.08.006

[pbi13326-bib-0006] Chen, Y.S. , Lo, S.F. , Sun, P.K. , Lu, C.A. , Ho, T.H.D. and Yu, S.M. (2015) A late embryogenesis abundant protein HVA 1 regulated by an inducible promoter enhances root growth and abiotic stress tolerance in rice without yield penalty. Plant Biotechnol. J. 13, 105–116. 25200982 10.1111/pbi.12241

[pbi13326-bib-0007] Collings, D.A. , Gebbie, L.K. , Howles, P.A. , Hurley, U.A. , Birch, R.J. , Cork, A.H. , Hocart, C.H. *et al*. (2008) Arabidopsis dynamin‐like protein DRP1A: a null mutant with widespread defects in endocytosis, cellulose synthesis, cytokinesis, and cell expansion. J. Exp. Bot. 59, 361–376. 18256049 10.1093/jxb/erm324

[pbi13326-bib-0008] Dure, L. , Greenway, S.C. and Galau, G.A. (1981) Developmental biochemistry of cottonseed embryogenesis and germination: changing messenger ribonucleic acid populations as shown by in vitro and in vivo protein synthesis. Biochemistry, 20, 4162–4168. 7284317 10.1021/bi00517a033

[pbi13326-bib-0009] El‐Sharkawy, I. , Mila, I. , Bouzayen, M. and Jayasankar, S. (2010) Regulation of two germin‐like protein genes during plum fruit development. J. Exp. Bot. 61, 1761–1770. 20202999 10.1093/jxb/erq043 PMC2852666

[pbi13326-bib-0010] Fanourakis, N.E. and Tzifaki, E.E. (1992) Correlated inheritance of fruit neck with fruit length and linkage relations with 10 other characteristics of cucumber. Euphytica, 65, 71–77.

[pbi13326-bib-0011] Gu, X. , Fang, X. and Han, X. (1994) Preliminary study of fruit neck length inheritance in cucumber. China Vegetable, 2, 33–34. (In Chinese)

[pbi13326-bib-0012] Hundertmark, M. and Hincha, D.K. (2008) LEA (late embryogenesis abundant) proteins and their encoding genes in *Arabidopsis thaliana* . BMC Genom. 9, 118. 10.1186/1471-2164-9-118 PMC2292704 18318901

[pbi13326-bib-0013] Kang, B.H. , Busse, J.S. and Bednarek, S.Y. (2003) Members of the Arabidopsis dynamin‐like gene family, ADL1, are essential for plant cytokinesis and polarized cell growth. Plant Cell, 15, 899–913. 12671086 10.1105/tpc.009670 PMC524700

[pbi13326-bib-0014] Kim, H.J. , Pesacreta, T.C. and Triplett, B.A. .(2004) Cotton‐fiber germin‐like protein. II: Immunolocalization, purification, and functional analysis. Planta, 218, 525–535. 14634817 10.1007/s00425-003-1134-0

[pbi13326-bib-0015] Kosambi, D.D. (1944) The estimation of map distance from recombination values. Annals of Eugenics, 12, 172–175.

[pbi13326-bib-0016] Kumar, S. , Stecher, G. , Li, M. , Knyaz, C. and Tamura, K. (2018) MEGA X: molecular evolutionary genetics analysis across computing platforms. Mol. Biol. Evol. 35, 1547–1549. 29722887 10.1093/molbev/msy096 PMC5967553

[pbi13326-bib-0017] Langmead, B. and Salzberg, S.L. (2012) Fast gapped‐read alignment with Bowtie 2. Nat. Methods 9, 357–359. 22388286 10.1038/nmeth.1923 PMC3322381

[pbi13326-bib-0018] Li, H. and Durbin, R. (2009) Fast and accurate short read alignment with Burrows‐Wheeler transform. Bioinformatics, 25, 1754–1760. 19451168 10.1093/bioinformatics/btp324 PMC2705234

[pbi13326-bib-0019] Liu, Q. (2018) Inheritance and QTL mapping of fruit length and fruit neck length in cucumber (*Cucumis sativus* L.). Master thesis, Yangzhou University, Yangzhou, Jiangsu, China.

[pbi13326-bib-0020] Livak, K. and Schmittgen, T. (2001) Analysis of relative gene expression data using real‐time quantitative PCR and the 2^−Δ ΔCt^ method. Methods 25, 402–408. 11846609 10.1006/meth.2001.1262

[pbi13326-bib-0021] Lu, H. , Lin, T. , Klein, J. , Wang, S. , Qi, J. , Zhou, Q. , Sun, J. *et al*. (2014) QTL‐seq identifies an early flowering QTL located near *Flowering Locus T* in cucumber. Theor. Appl. Genet. 127, 1491–1499. 24845123 10.1007/s00122-014-2313-z

[pbi13326-bib-0022] Ma, J. , Si, L. and Tian, Y. (2010) Mixed major gene and polygene inheritance analysis of fruit stalk length in cucumber. Acta Agriculturae Boreali‐Occidentalis Sinica 19, 161–165.

[pbi13326-bib-0023] Mathieu, M. , Neutelings, G. , Hawkins, S. , Grenier, E. and David, H. (2003) Cloning of a pine germin‐like protein (GLP) gene promoter and analysis of its activity in transgenic tobacco Bright Yellow 2 cells. Physiol. Plant. 117, 425–434. 12654044 10.1034/j.1399-3054.2003.00050.x

[pbi13326-bib-0024] Murray, M. and Thompson, W.F. (1980) Rapid isolation of high molecular weight plant DNA. Nucleic Acids Res. 8, 4321–4326. 7433111 10.1093/nar/8.19.4321 PMC324241

[pbi13326-bib-0025] Olvera‐Carrillo, Y. , Luis Reyes, J. and Covarrubias, A. (2011) Late embryogenesis abundant proteins: versatile players in the plant adaptation to water limiting environments. Plant Signal. Behav. 6, 586–589. 21447997 10.4161/psb.6.4.15042 PMC3142399

[pbi13326-bib-0026] Pan, Y. , Liang, X. , Gao, M. , Liu, H. , Meng, H. , Weng, Y. and Cheng, Z. (2017) Round fruit shape in WI7239 cucumber is controlled by two interacting quantitative trait loci with one putatively encoding a tomato SUN homolog. Theor. Appl. Genet. 130, 573–586. 27915454 10.1007/s00122-016-2836-6

[pbi13326-bib-0027] Pandey, M.K. , Khan, A.W. , Singh, V.K. , Vishwakarma, M.K. , Shasidhar, Y. , Kumar, V. , Garg, V. *et al*. (2017) QTL‐seq approach identified genomic regions and diagnostic markers for rust and late leaf spot resistance in groundnut (*Arachis hypogaea* L.). Plant Biotechnol. J. 15, 927–941. 28028892 10.1111/pbi.12686 PMC5506652

[pbi13326-bib-0028] Qi, J. , Liu, X. , Shen, D. , Miao, H. , Xie, B. , Li, X. , Zeng, P. *et al*. (2013) A genomic variation map provides insights into the genetic basis of cucumber domestication and diversity. Nat. Genet. 45, 1510–1515. 24141363 10.1038/ng.2801

[pbi13326-bib-0029] Riewe, D. , Jeon, H.J. , Lisec, J. , Heuermann, M.C. , Schmeichel, J. , Seyfarth, M. , Meyer, R.C. *et al*. (2016) A naturally occurring promoter polymorphism of the Arabidopsis FUM2 gene causes expression variation, and is associated with metabolic and growth traits. Plant J. 88, 826–838. 27520391 10.1111/tpj.13303

[pbi13326-bib-0030] Salleh, F.M. , Evans, K. , Goodall, B. , Machin, H. , Mowla, S.B. , Mur, L.A. , Runions, J. *et al*. (2012) A novel function for a redox‐related LEA protein (SAG21/AtLEA5) in root development and biotic stress responses. Plant Cell Environ. 35, 418–429. 21736589 10.1111/j.1365-3040.2011.02394.x

[pbi13326-bib-0031] Singh, V.K. , Khan, A.W. , Jaganathan, D. , Thudi, M. , Roorkiwal, M. , Takagi, H. , Garg, V. *et al*. (2016) QTL‐seq for rapid identification of candidate genes for 100‐seed weight and root/total plant dry weight ratio under rainfed conditions in chickpea. Plant Biotechnol. J. 14, 2110–2119. 27107184 10.1111/pbi.12567 PMC5095801

[pbi13326-bib-0032] Takagi, H. , Abe, A. , Yoshida, K. , Kosugi, S. , Natsume, S. , Mitsuoka, C. , Uemura, A. *et al*. (2013) QTL‐seq: rapid mapping of quantitative trait loci in rice by whole genome resequencing of DNA from two bulked populations. Plant J. 74, 174–183. 23289725 10.1111/tpj.12105

[pbi13326-bib-0033] Wang, G. , Qin, Z. , Zhou, X. and Zhao, Z. (2008) Mapping Quantitative Trait Loci Effecting Cucumber Carpopodium Length Using Simple Sequence Repeat Markers. Acta Horticulturae Sinica 35, 543–546.

[pbi13326-bib-0034] Wang, M. , Liu, S. , Zhang, S. , Miao, H. , Wang, Y. , Tian, G. , Lu, H. *et al*. (2014) Quantitative trait loci associated with fruit length and stalk length in cucumber using RIL population. Acta Botanica Boreali‐Occidentalia Sinica, 34, 1764–1770.

[pbi13326-bib-0035] Wang, X. , Bao, K. , Reddy, U.K. , Bai, Y. , Hammar, S.A. , Jiao, C. , Wehner, T. *et al*. (2018) The USDA cucumber (*Cucumis sativus* L.) collection: genetic diversity, population structure, genome‐wide association studies, and core collection development. Horticulture Research 5, 64. 30302260 10.1038/s41438-018-0080-8 PMC6165849

[pbi13326-bib-0036] Weng, Y. , Colle, M. , Wang, Y. , Yang, L. , Rubinstein, M. , Sherman, A. , Ophir, R. *et al*. (2015) QTL mapping in multiple populations and development stages reveals dynamic quantitative trait loci for fruit size in cucumbers of different market classes. Theor. Appl. Genet. 128, 1747–1763. 26048092 10.1007/s00122-015-2544-7

[pbi13326-bib-0037] Win, K.T. , Vegas, J. , Zhang, C. , Song, K. and Lee, S. (2017) QTL mapping for downy mildew resistance in cucumber via bulked segregant analysis using next‐generation sequencing and conventional methods. Theor. Appl. Genet. 130, 199–211. 27714417 10.1007/s00122-016-2806-z

[pbi13326-bib-0038] Xiong, G. , Li, R. , Qian, Q. , Song, X. , Liu, X. , Yu, Y. , Zeng, D. *et al*. (2010) The rice dynamin‐related protein DRP2B mediates membrane trafficking, and thereby plays a critical role in secondary cell wall cellulose biosynthesis. Plant J. 64, 56–70. 20663087 10.1111/j.1365-313X.2010.04308.x

[pbi13326-bib-0039] Xu, X. , Lu, L. , Zhu, B. , Xu, Q. , Qi, X. and Chen, X. (2015) QTL mapping of cucumber fruit flesh thickness by SLAF‐seq. Sci. Rep. 5, 15829. 26508560 10.1038/srep15829 PMC4623748

[pbi13326-bib-0040] Xu, X. , Ji, J. , Xu, Q. , Qi, X. , Weng, Y. and Chen, X. (2018) The major‐effect quantitative trait locus *CsARN 6.1* encodes an AAA ATP ase domain‐containing protein that is associated with waterlogging stress tolerance by promoting adventitious root formation. Plant J. 93, 917–930. 29315927 10.1111/tpj.13819

[pbi13326-bib-0042] Yuan, X.J. , Li, X.Z. , Pan, J.S. , Wang, G. , Jiang, S. , Li, X.H. , Deng, L. *et al*. (2008) Genetic linkage map construction and location of QTLs for fruit‐related traits in cucumber. Plant Breed. 127, 180–188.

[pbi13326-bib-0043] Zhao, J. , Li, Y. , Ding, L. , Yan, S. , Liu, M. , Jiang, L. , Zhao, W. *et al*. (2015) Phloem transcriptome signatures underpin the physiological differentiation of the pedicel, stalk and fruit of cucumber (*Cucumis sativus* L.). Plant Cell Physiol. 57, 19–34. 26568324 10.1093/pcp/pcv168

[pbi13326-bib-0044] Zhou, X.Y. , Qin, Z.W. and Wang, X.G. (2005) Study of the market characters of cucumber (*Cucumis sativus* L.) germplasm. J. Northeast Agric. Univ. 36, 707–713. (In Chinese with English abstract)

