# Supplementary material for: The major‐effect quantitative trait locus Fnl7.1 encodes a late embryogenesis abundant protein associated with fruit neck length in cucumber
Source: Plant Biotechnol J. 2020 Jan 24;18(7):1598–609. doi: 10.1111/pbi.13326 (PMC7292543; doi:10.1111/pbi.13326)
Supplement: Supplementary file 1 — Figure S1 Alignment of the 14.1‐kb genomic DNA sequences from Jin5‐508 and YN. [file PBI-18-1598-s002.pdf]

Fig.S1 Alignment of the 14.1-kb genomic DNA sequences from Jin5-508 (upper) and YN (lower). The pair-wise comparison was performed by using the ApE (v2.0.60) software with default parameters. The software is accessible from http://jorgensen.biology.utah.edu/wayned/ape/. The variants (SNPs and InDels) were highlighted in red. The blue nucleotide sequences are the three exons of CsGy7G014720 (CsFnl7.1) while the orange nucleotide sequences are the the exon of CsGy7G014730.

```
   1 TGCAAAACCAAGGCGTTAATTTCTCATAGCAGACAACAAACAAACAGAAACTTTTTGAATAATGTGACAAATAATATTATCATAAGAAGAGCTATATCACTTATTAGTTCACAACCTAAATACCTTTGTTAAGATGTAAAATATTGAAAA 150
   1 TGCAAAACCAAGGCGTTAATTTCTCATAGCAGACAACAAACAAACAGAAACTTTTTGAATAATGTGACAAATAATATTATCATAAGAAGAGCTATATCACTTATTAGTTCACAACCTAAATACCTTTGTTAAGATGTAAAATATTGAAAA 150

 151 ATAATATTATCATAGAGTTCAAATGACTTTTTTTTCATCTTCGCTTTCGCGTTTCTTAGTAAAGTGACATCTCACAGAAAAGAAAGTAAAAGAAATCTGTAATTACCAATATGGATTCATTTAAAGAATGAACTGTTGTTTAAACTCTCT 300
 151 ATAA-ATTA----AGAGTTCAAATGACTTTTTTTTCATCTTCGCTTTCGCGTTTCTTAGTAAAGTGACATCTCACAGAAAAGAAAGTAAAAAGAATCTGTAATTACCAATATGGATTCATTTAAAGAATGAACTGTTCTTTAAACTCTCT 295

 301 TCTATAGATTATATAACGTTCCTATTTATAATTATTAATAACTGAGTAATTAACAATCATGTTAAACTAATCTTTCAAAATTAATAGAATTGAAATGAATTGAAGATTTTGTATTTAAAAGAATCACACAAACACATGTACATTATTCCA 450
 296 TCTATAGATTATATAACGTTCGTATTTATAATTATTAATAACTGAGTAATTAACAATCATGTTAAACTAATCTTTCAAAATTAATAGAATTGAAATGAATTGAAGATTTTGTATTTAAAAGAATCACACGAACACATGTACATTATTTCA 445

 451 TGGGTTAACCTCTTCATCAAGTTCCAAGTGTCAGTTAAGCATATTGCATGGCCATTTATTAGTTTTCATATCCACATGTATATTCCTCCATCCATTCTCTAAAAGCTTCTCATTCTCCTCTCAACACTTGTAAATCCCTTTCTTCTCTCA 600
 446 TGGCTTAACCTCTTCATC-AGTTCCAAGTGTCAGTTAAGCATATTGCATGGCCATTTATTAGTTTTCATATCCACATGTATATTCCTCCATCCATTCTCTAAAAGCTTCTCATTCTCCTCTCAACACTTGTAAATCCCTTTCTTCTCTCA 594

 601 CAATCTCATGTGTGTATATATATATTATACACAAACACATTAAATTAATTATTACTTCCAACAACAAAAGAAAAGAAAAAAAAAAGAAATGAGTCAAGGACAGCCTCAAAGGCCTGCCGGAGATAATTACAAGGATCCAGTCAAATATGGC 750
 595 CAATCTCATGTGTGTATATATATATTATACACAAACACATTAAATTAATTATTACTTCCAACAACAAAAGAAAAGAAAAAAAAAAGAAATGAGTCAAGGACAGCCTCAAAGGCCTGCCGGAGATAATTACAAGGATCCAGTCAAATATGGC 744

 751 GACGTTTTTGACGTCTCCGGCGAGTTGGCCTCCAAGCCCATTGCCCCTCGTGATGCTGCCAGCTTGCAGGCCGCTGAGAATATAATTCTCGGCCAGACTCCTCGTGGCGGCCCTGCTGCGGTTATGCAGTCCGCTGCCAATCTCAACGAA 900
 745 GACGTTTTTGACGTCTCCGGCGAGTTGGCCTCCAAGCCCATTGCCCCTCGTGATGCTGCCAGCTTGCAGGCCGCTGAGAATATAATTCTCGGCCAGACTCCTCGTGGCGGCCCTGCTGCGGTTATGCAGTCCGCTGCCAATCTCAACGAA 894

 901 CGTGCTGGGCTTGTTGGTCACTCCGACGTGACCGATGTTGCTCTAAACGAAGGTGTGAACGTGACTGAAACTGTGGTCGACGGTCAACGATTCGTGGTTGAGTCGGTGGGCGGACAGGTAAATTTTAGGTTTTGATTTTAATTTTAAATA 1050
 895 CGTGCTGGGCTTGTTGGTCACTCCGACGTGACCGATGTTGCTCTAAACGAAGGTGTGAACGTGACTGAAACTGTGGTCGACGGTCAACGATTCGTGGTTGAGTCGGTGGGCGGACAGGTAAATTTTAGGTTTTGATTTTAATTTTAAATA 1044

1051 TTTGATTAACGTGATTTTAATTATGTGTATTAACATATTGATACGATTATTTTCTGAAGAAATTTAATTGGATATGGTTTGCAGTGTTATGGGCAAAATATTAAAAAAAAAAAAAAAAGGAAAATGGAAGTTTATGATGAGATTAATTGTC 1200
1045 TTTGATTAACGTGATTTTAATTATGTGTATTAACATATTGATACGATGATTTTCTGAAGAAATTTAATTGGATATGGTTTGCAGTGTTATGGGCAAAATATTAAAAAAAAAAAAAAAAGGAAAATGGAAGTTTATGATGAGATTAATTGTC 1194

1201 AAAGAGAGAAAATTGGCCAAAATGTTTTAAATTTTGAGCTAGTGATGAAAAGAAATCAAGTTCATCGATTGATAGTGATACCTTTTTCCCCTTAAGATTGTGGGACGTTTTGTCCAACCAAACGTTCCAATGAGATCTCCAGGAGCAACA 1350
1195 AAAGAGAGAAAATTGGCCAAAATGTTTTAAATTTTGAGCTAGTGATGAAAAGAAATCAAGTTCATCGATTGATAGTGATACCTTTTTCCCCTTAAGATTGTGGGACGTTTTGTCCAACCAAATGTTCCAATGAGATCTCCAGGAGCAACA 1344

1351 CTCGACAATGATTCAATCAGCATTGGAGAAGCTTTAGAAGCCAGCGCAGTCAGTGCGGGAGACAAGCCGATTAATCAAAGCGACGCGGCCGCAATCCAAGCGGCCGAGGTTCGTGCGACCGGGGAAAACAATATAATCCCAGGTGGCATT 1500
1345 CTCGACAATGATTCAATCAGCATTGGAGAAGCTTTAGAAGCCAGCGCAGTCAGTGCGGGAGACAAGCCGATTAATCAAAGCGACGCGGCCGCAATCCAAGCGGCCGAGGTTCGTGCGACCGGGGAAAACAATATAATACCAGGTGGCATT 1494

1501 GGGGCGGAGGCACAATCGGCTGCCACCCTCAACACTCGAGTCATGCTGAACGAGAATAAGACAACACTTGGCGACATTCTAACCGTAAGAGAACTTGTTACCAATTCAAACCTGCTTGTTTTTAGGCGATTAATGCCTTAACGTAGTATT 1650
1495 GGGGCGGAGGCACAATCGGCTGCCACCCTCAACACTCGAGTCATGCTGAACGAGAATAAGACAACACTTGGCGACATTCTAACCGTAAGAGAACTTGTTACCAATTCAAACCTGCTTGTTTTTAGGCGATTAATGCCTTAACGTAGTATT 1644

1651 GAAGTATGTTTGATAAATTTGATAGGATGCGACACGAAAGTTGCCAGGAGATAAGACGGTGACAAAGGAAGACGCAGAGAGGGTGATAAGTGCGGAGATAAGGAACGACCTAAAAATGGCTACTACCCCGGGAGGGGTGGCAGCGTCAGT 1800
1645 GAAGTATGTTTGATAAATTTGATAGGATGCGACACGAAAGTTGCCAGGAGATAAGACGGTGACAAAGGAAGACGCAGAGAGAGTGATAAGTGCGGAGATAAGGAACGACCTAAAAATGGCTACTACCCCGGGAGGGGTGGCAGCGTCAGT 1794

1801 GGCAGCCGCGGCAAGGCTTAACCAACAACAGGGGTAGCATAGTGTATTTTGATCATATAGTAGCAAGTTCCTCCTTCTCTGCTTTATTTTATGGATGTCAAGAAGTTTTCAAGTGGTTGTTAGGAGTACTGTGTTGTTATCTTTTTTGTT 1950
1795 GGCAGCCGCGGCAAGGCTTAACCAACAACAGGGGTAGCATAGTGTATTTTGATCATATAGTAGCAAGTTCCTCCTTCTCTGCTTTATTTTATGGATGTCAAGAAGTTTTCAAGTGGTTGTTAGGAGTACTGTGTTGTTATCTTTTTTGTT 1944

1951 TAGGAGGTGTGTGTTTAATTTCTAGTGTGTAGGGTTATATTTTATAGTTAGACTTTTAGTAGTGGTTTTGAGAGGATATATATATGTATCTCTTTAATGGAACCAAGTTATTGTACATCTAAGATTATTCCAAATAAGCTTAAAAAAGAT 2100
1945 TAGGAGGTGTGTGTTTAATTTCTAGTGTGTAGGGTTATATTTTATAGTTAGACTTTTAGTAGTGGTTTTGAGAGGATATATATATGTATCTCTTTAATGGAACCAAGTTATTGTACATCTAAGATTATTCCAAATAAGCTTAAAAAAGAT 2094

2101 GCATGGGATTAATTAACTGACAACTATAAATTGAGTGTACTTAGAATGCATTCTCAATTTTTAATTTAAAAATGAGTTGTTTTTTCTTTTTCTAAAAAAAGAGTTATTTGGCCACTGCTAAAAGTTAAAAACATTTTTTAAAGTTAATCT 2250
2095 GCATGGGATTAATTAACTGACAACTATAAATTGAGTGTACTTAGAATGCATTCTCAATTTTTAATTTAAAAATGAGTTGTTTTTTCTTTTTCTAAAAAAAGAGTTATTTGGCCACTGCTAAAAGTTAAAAACATTTTTTAAAGTTAATCT 2244

2251 TAACCATGTAAGCAAATTTTAATTTGTATGAGAATATTAGTATTCTAAAAAAAGAGTGAAATCCACCAAACCCTCAATTTAGCTCATTTAAATTTTTTCTTTCTCCGGAAACAAAAATAGAGAATTTTAAAAAATATATATATATATGAG 2400
2245 TAACCATGTAAGCAAATTTTAATTTGTATGAGAATATTAGTATTCTAAAAAAAGAGTGAAATCCACCAAACCCTCAATTTAGCTCATTTAAATTTTTTCTTTCTCCGGAAACAAAAATAGAGAATTTTAAAAAATATATATATATATGAG 2394

2401 TGTTTATAAGGAGTTTATTTACTTTTCTTTTATTTTTTTAATCAAGGTGTTTAAATGAGAATGTACTTTTGATTCTCTTTAATCATTTCAAATTGGCCAGAAATAATTTTCCATGAAGAAAATGATATACATTATAAAATTTTGGCATAC 2550
2395 TGTTTATAAGGAGTTTATTTACTTTTCTTTTATTTTTTTAATCAAGGTGTTTAAATGAGAATGTACTTTTGATTCTCTTTAATCATTTCAAATTGGCCAGAAATAATTTTCCATGAAGAAAATGATATACATTATAAAATTTTGGCATAC 2544

2551 ATATAATGTGTGATTGGACTGTAGGCTCATTGCTGGGCTAATTTAGTGGAGATCCATATTTGATGATTTTGATGATAACCATTCGTATCCATATTCATATTAATCATATTAGTGGAGAATTTTTTAACTGTGCTTTCTACTTCACTATAT 2700
2545 ATATAATGTGTGATTGGACTGTAGGCTCATTGCTGGGCTAATTTAGTGGAGATCCATATTTGATGATTTTGATGATAACCATTCGTATCCATATTCATATTAATCATATTAGTGGAGAATTTTTTAACTGTGCTTTCTACTTCACTATAT 2694

2701 ATATAAATATTTTCGTGTTTTCAACTTGACTATATTACATATATATCCAAACATATTTCTTTAAAAAAAATTGTGATACACAGGAGATCAAAAGTGGAGTGAATGTTATTGGGAGTGAGATTTAAGAGTGGGGAGCTAATTTTAACAAAGT 2850
2695 ATATAAATATTTTCGTGTTTTCAACTTGACTATATTACATATATATCCAAACATATTTCTTTAAAAAAAATTGTGATACACAGGAGATCAAAAGTGGAGTGAATGTTATTGGGAGTGAGATTTAAGAGTGGGGAGCTAATTTTAACAAAGT 2844

2851 ACACAAAATATATAATAATTAAAGTATAATATAATTGGCATAACCTTTTTGTAGATTCCCATTTCAAAAGACACAAAAATTGTAGAGTCAAATTATGAGTGCGTTTGCAAAGTGTGCTTTGTTGTTTCTTGTTTCCATGGAGAGAAAAAC 3000
2845 ACACAAAATATATAATAATTAAAGTATAATATAATTGGCATAACCTTTTTGTAGATTCCCATTTCAAAAGACACAAAAATTGTAGAGTCAAATTATGAGTGCGTTTGCAAAGTGTGCTTTGTTGTTTCTTGTTTCCATGGAGAGAAAAAC 2994

3001 AAATAACTTACAGCTAACATCTAAGATCTTGATTTCATATAATATTCTCACTATCTTTTTCTGTGCTTTTATAAATTCTATTAAAAAAAACTGTTCTTTCTGGCTCCTTGGAAACTTCTTAGTTTTGTTGTGTTTGTATATATACATATA 3150
2995 AAATAACTTACAGCTAACATCTAAGATCTTGATTTCATATAATATTCTCACTATCTTTTTCTGTGCTTTTATAAATTCTATTAAAAAAAACTGTTCTTTCTGGCTCCTTGGAAACTTCTTAGTTTTGTTGTGTTTGTATATATACATATA 3144
```

```
              *         *         *         *         *         *         *         *         *         *         *         *         *         *         *
3151 TATTATTCTCTTAGGGGCTTTAGCTTAGCTAATATGAATCTTAATCATTTAGGTTTTGGCAAATGCATTATCAGATTTGGTCATACATGTTCTTCTTTAAATTAATTAAATTATTATTATTATATATGTTTGTATATTTGCCCATAAAAC 3300
     ||||||||||||||||||||||||||||||||||||||||||||||||||||||||||||||||||||||||||||||||||||||||||||#|||||||||||||||||||||||||||||||||||||||||||||||||||||||
3145 TATTATTCTCTTAGGGGCTTTAGCTTAGCTAATATGAATCTTAATCATTTAGGTTTTGGCAAATGCATTATCAGATTTGGTCATACATGTTCTTTTTTAAATTAATTAAATTATTATTATTATATATGTTTGTATATTTGCCCATAAAAC 3294
          *         *         *         *         *         *         *         *         *         *         *         *         *         *         *

              *         *         *         *         *         *         *         *         *         *         *         *         *         *         *
3301 TTATAGACAACATAATATAAATGTTTTAGTCATGTCTTTTATCTCCAATTAGGTTATTCACGAATAACGCTAGCTCACATAATGATAAGAATAATAAGTTTACTAATGATACAATAATGTAAACGCAAGGAAAACAAAATGTGTGGAAAA 3450
     ||||||||||||||||||||||||||||||||||||||||||||||||||||||||||||||||||||||||||||||||||||||||||||||||||||||||||||||||||||||||||||||||||||||||||||||||||||
3295 TTATAGACAACATAATATAAATGTTTTAGTCATGTCTTTTATCTCCAATTAGGTTATTCACGAATAACGCTAGCTCACATAATGATAAGAATAATAAGTTTACTAATGATACAATAATGTAAACGCAAGGAAAACAAAATGTGTGGAAAA 3444
          *         *         *         *         *         *         *         *         *         *         *         *         *         *         *

              *         *         *         *         *         *         *         *         *         *         *         *         *         *         *
3451 GTAGTGTTTGTAAATAAATCTATTATAAATACAATATTTTGCATTCTATTTTGTTGTCAACATTATAGAAGTGATTAACTGATTAAAATTCAAGTTAATTTTTCAATAAAATTATGAGAAAAAGTCAAAATTACACAAACAAAAAAAAAA 3600
     ||||||||||||||||||||||||||||||||||||||||||||||||||||||||||||||||||||||||||||||||||||||||||||||||||||||||||||||||||||||||||||||||||||||||||||||||||||
3445 GTAGTGTTTGTAAATAAATCTATTATAAATACAATATTTTGCATTCTATTTTGTTGTCAACATTATAGAAGTGATTAACTGATTAAAATTCAAGTTAATTTTTCAATAAAATTATGAGAAAAAGTCAAAATTACACAAACAAAAAAAAAA 3594
          *         *         *         *         *         *         *         *         *         *         *         *         *         *         *

              *         *         *         *         *         *         *         *         *         *         *         *         *         *         *
3601 GTTTTTCTTAGGAAGTTTGATTAAGAATTTAATAACAGATAAACTACTAGTATAGTTTAACTAACATGAAAATCATTCAAATCATGGTGTAGAAATAAGGATAATTAAATAAATATATCAATTTAAAGCAATTCTAACTATGCAATTCTA 3750
     ||||||||||||||||||||||||||||||||||||||||||||||||||||||||||||||||||||||||||||||||||||||||||||||||||||||||||||||||||||||||||||||||||||||||||||||||||||
3595 GTTTTTCTTAGGAAGTTTGATTAAGAATTTAATAACAGATAAACTACTAGTATAGTTTAACTAACATGAAAATCATTCAAATCATGGTGTAGAAATAAGGATAATTAAATAAATATATCAATTTAAAGCAATTCTAACTATGCAATTCTA 3744
          *         *         *         *         *         *         *         *         *         *         *         *         *         *         *

              *         *         *         *         *         *         *         *         *         *         *         *         *         *         *
3751 AATTAGGTGATCTTCCTTATCTAATTTTTTTAAATTATAATTCACAATTTATTTTATGGATGTCAACATACTGATTTTGTTCTCCTTTTTCTGTCTTTTTTTTTTCTTTGGACATATTAGATTATGTTCTTTAGTAATTAGATAATTAAA 3900
     ||||||||||||||||||||||||||||||||||||||||||||||||||||||||||||||||||||||||||||||||||||||||||||||||||||||||||||||||||||||||||||||||||||||||||||||||||||
3745 AATTAGGTGATCTTCCTTATCTAATTTTTTTAAATTATAATTCACAATTTATTTTATGGATGTCAACATACTGATTTTGTTCTCCTTTTTCTGTCTTTTTTTTTTCTTTGGACATATTAGATTATGTTCTTTAGTAATTAGATAATTAAA 3894
          *         *         *         *         *         *         *         *         *         *         *         *         *         *         *

              *         *         *         *         *         *         *         *         *         *         *         *         *         *         *
3901 ATAAATAAACATTATATATATATATATATATATATATATATATATATATATATATATATATATATTGAAGCATAATTTTGTAAGGTGTTGCTTATCAATAAAAACAATGGAAGACAGTCCCTATAATTAGGAGAGAAAAGACTTCCCTTTTCGTGT 4050
     ||||||||||||||||||||||||||||||||||||||||||||||||||||||||||||||||||||||||||||||||||||||||||||||||||||||||||||||||||||||||||||||||||||||||||||||||||||
3895 ATAAATAAACATTATATATATATATATATATATATATATATATATATATATATATATATATATATTGAAGCATAATTTTGTAAGGTGTTGCTTATCAATAAAAACAATGGAAGACAGTCCCTATAATTAGGAGAGAAAAGACTTCCCTTTTCGTGT 4044
          *         *         *         *         *         *         *         *         *         *         *         *         *         *         *

              *         *         *         *         *         *         *         *         *         *         *         *         *         *         *
4051 TTTATTTATTAATATTTTTTGTCTTTAAAATTAGAGTTTCAAATAATATATATACCCTTCAATTATTTGACAAAATTTTATAATATATATTTGACAAAACTCTGAGGTGGCATATCAAATGTATGTTTCACTTAGGCACTTTCAATTATG 4200
     ||||||||||||||||||||||||||||||||||||||||||||||||||||||||||||||||||||||||||||||||||||||||||||||||||||||||||||||||||||||||||||||||||||||||||||||||||||
4045 TTTATTTATTAATATTTTTTGTCTTTAAAATTAGAGTTTCAAATAATATATATACCCTTCAATTATTTGACAAAATTTTATAATATATATTTGACAAAACTCTGAGGTGGCATATCAAATGTATGTTTCACTTAGGCACTTTCAATTATG 4194
          *         *         *         *         *         *         *         *         *         *         *         *         *         *         *

              *         *         *         *         *         *         *         *         *         *         *         *         *         *         *
4201 GAATGGGATATGATCTATTTATAACTTTGTTTTTGCTAAAAAGAAAAATAACAACTGTATAGTTTTTCAATTTAATAAAATTAGCATAAGAGTATTTGAACCCATATGTGGATGAGTTCAAATTGAATCTTACTCTACCTAAGTTCAACA 4350
     ||||||||||||||||||||||||||||||||||||||||||||||||||||||||||||||||||||||||||||||||||||||||||||||||||||||||||||||||||||||||||||||||||||||||||||||||||||
4195 GAATGGGATATGATCTATTTATAACTTTGTTTTTGCTAAAAAGAAAAATAACAACTGTATAGTTTTTCAATTTAATAAAATTAGCATAAGAGTATTTGAACCCATATGTGGATGAGTTCAAATTGAATCTTACTCTACCTAAGTTCAACA 4344
          *         *         *         *         *         *         *         *         *         *         *         *         *         *         *

              *         *         *         *         *         *         *         *         *         *         *         *         *         *         *
4351 TTTTTGGTCCAAATTTCTAGAAATGGATTCAAATTACACAATCTTTTGGTAATCTTTTGGTAATTGGTTAAGTCATGTACACATCTTCATTAAATAAAAATTAAGATTTCCATCATTCAATTTTAAGAATCAATATATTGAAATAAATTT 4500
     ||||||||||||||||||||||||||||||||||||||||||||||||||||||||||||||||||||||||||||||||||||||||||||||||||||||||||||||||||||||||||||||||||||||||||||||||||||
4345 TTTTTGGTCCAAATTTCTAGAAATGGATTCAAATTACACAATCTTTTGGTAATCTTTTGGTAATTGGTTAAGTCATGTACACATCTTCATTAAATAAAAATTAAGATTTCCATCATTCAATTTTAAGAATCAATATATTGAAATAAATTT 4494
          *         *         *         *         *         *         *         *         *         *         *         *         *         *         *

              *         *         *         *         *         *         *         *         *         *         *         *         *         *         *
4501 TCTTTTTTTTCCTTCCATATAAAGTGCAGGTATATATTAAAGGTTTTAATATTTGTAATTTTGTATGGTCTATCGTGTTTATATATATATATATATACTCTTTTCAAAATCAATACTAAAATATTTAAAAAAAAACATGATAGTACAATGAT 4650
     ||||||||||||||||||||||||||||||||||||||||||||||||||||||||||||||||||||||||||||||||||||||||||||||||||||||||||||||||||||||||||||||||||||||||||||||||||||
4495 TCTTTTTTTTCCTTCCATATAAAGTGCAGGTATATATTAAAGGTTTTAATATTTGTAATTTTGTATGGTCTATCGTGTTTATATATATATATATATACTCTTTTCAAAATCAATACTAAAATATTTAAAAAAAAACATGATAGTACAATGAT 4644
          *         *         *         *         *         *         *         *         *         *         *         *         *         *         *

              *         *         *         *         *         *         *         *         *         *         *         *         *         *         *
4651 GCATAAGAAAATTGATTAAAATGGGAAAAAAAAATAGAAATAGAAAATATGGATAAAATAAAAGAGATCCAAAGAAGATACAATATAATATTTATTAAAACATATTTATATATAGCAAAAAAAATAGAAAATATAACAAAATTTTGGAGCA 4800
     ||||||||||||||||||||||||||||||||||||||||||||||||||||||||||||||||||||||||||||||||||||||||||||||||||||||||||||||||||||||||||||||||||||||||||||||||||||
4645 GCATAAGAAAATTGATTAAAATGGGAAAAAAAAATAGAAATAGAAAATATGGATAAAATAAAAGAGATCCAAAGAAGATACAATATAATATTTATTAAAACATATTTATATATAGCAAAAAAAATAGAAAATATAACAAAATTTTGGAGCA 4794
          *         *         *         *         *         *         *         *         *         *         *         *         *         *         *

              *         *         *         *         *         *         *         *         *         *         *         *         *         *         *
4801 ATATAACATTGGTATGACATAAATATGAGGTTAATAAGACTATTAATGATAAAGTTCATTAAGAAAAATGTAAGAAGAGAATTATATTGAAAGATAGAGGGTAATAGTGGAAGAAATGAGTAGGGAAGTATGGAGGGTGCTAAAAGTGAT 4950
     ||||||||||||||||||||||||||||||||||||||||||||||||||||||||||||||||||||||||||||||||||||||||||||||||||||||||||||||||||||||||||||||||||||||||||||||||||||
4795 ATATAACATTGGTATGACATAAATATGAGGTTAATAAGACTATTAATGATAAAGTTCATTAAGAAAAATGTAAGAAGAGAATTATATTGAAAGATAGAGGGTAATAGTGGAAGAAATGAGTAGGGAAGTATGGAGGGTGCTAAAAGTGAT 4944
          *         *         *         *         *         *         *         *         *         *         *         *         *         *         *

              *         *         *         *         *         *         *         *         *         *         *         *         *         *         *
4951 ACTTATGGTAAATATTGACTTTGATGAAATATTGTATAGAAAAGAATGGTATATGATATAATAACTTGAAAGTAAAAGTGGAGTGAAAGTACAATAATGGGAGAGATGTAGTAAGGTGACATTTGATTAATTTCAAAACACCAAAACTTT 5100
     ||||||||||||||||||||||||||||||||||||||||||||||||||||||||||||||||||||||||||||||||||||||||||||||||||||||||||||||||||||||||||||||||||||||||||||||||||||
4945 ACTTATGGTAAATATTGACTTTGATGAAATATTGTATAGAAAAGAATGGTATATGATATAATAACTTGAAAGTAAAAGTGGAGTGAAAGTACAATAATGGGAGAGATGTAGTAAGGTGACATTTGATTAATTTCAAAACACCAAAACTTT 5094
          *         *         *         *         *         *         *         *         *         *         *         *         *         *         *

              *         *         *         *         *         *         *         *         *         *         *         *         *         *         *
5101 TTTGTTGTTCCCTTTCCCTCTCATTTCTCACATCAATTCTGTTTATACAGTATTTTTTAGTACAATCCCTTTTTCAAGTCTCATCATTTCTACCTCATACATCACACAACTCAACAAAAAAACAAAATATATAGGTAACTAGATATCAAT 5250
     ||||||||||||||||||||||||||||||||||||||||||||||||||||||||||||||||||||||||||||||||||||||||||||||||||||||||||||||||||||||||||||||||||||||||||||||||||||
5095 TTTGTTGTTCCCTTTCCCTCTCATTTCTCACATCAATTCTGTTTATACAGTATTTTTTAGTACAATCCCTTTTTCAAGTCTCATCATTTCTACCTCATACATCACACAACTCAACAAAAAAACAAAATATATAGGTAACTAGATATCAAT 5244
          *         *         *         *         *         *         *         *         *         *         *         *         *         *         *

              *         *         *         *         *         *         *         *         *         *         *         *         *         *         *
5251 GATTGTTGACATCGAATTCACGATCTTATGATATAAAATAACTTATATCTATTTAATAATCATTTCATTTTTTTGTTTTTAGTTCTTGGAAATGAAAGAAGCTGTGAAATATGTGGCTAATTAATCTTAAATTTAAATTATATGCATGCC 5400
     ||||||||||||||||||||||||||||||||||||||||||||||||||||||||||||||||||||||||||||||||||||||||||||||||||||||||||||||||||||||||||||||||||||||||||||||||||||
5245 GATTGTTGACATCGAATTCACGATCTTATGATATAAAATAACTTATATCTATTTAATAATCATTTCATTTTTTTGTTTTTAGTTCTTGGAAATGAAAGAAGCTGTGAAATATGTGGCTAATTAATCTTAAATTTAAATTATATGCATGCC 5394
          *         *         *         *         *         *         *         *         *         *         *         *         *         *         *

              *         *         *         *         *         *         *         *         *         *         *         *         *         *         *
5401 TTTGTAGTATAAATTTTATGAAGTAATAATTATTTCAAGTGTTGGTTAGACTGTGGTAGAAAATAAAATCTACTTTCAAACTTCAAAATTAGGATGGTGTGAAAAAGATAACTTTGGTAGTCAAAATTAGTTGTTGGGTAAAAAGAGAGA 5550
     ||||||||||||||||||||||||||||||||||||||||||||||||||||||||||||||||||||||||||||||||||||||||||||||||||||||||||||||||||||||||||||||||||||||||||||||||||||
5395 TTTGTAGTATAAATTTTATGAAGTAATAATTATTTCAAGTGTTGGTTAGACTGTGGTAGAAAATAAAATCTACTTTCAAACTTCAAAATTAGGATGGTGTGAAAAAGATAACTTTGGTAGTCAAAATTAGTTGTTGGGTAAAAAGAGAGA 5544
          *         *         *         *         *         *         *         *         *         *         *         *         *         *         *

              *         *         *         *         *         *         *         *         *         *         *         *         *         *         *
5551 GCAATAACATTGAATTGAGCAATGGTGTTTTTGTTCTTTATTTAAACACATGTGATGTATTAGAACGAAAAAATATATATATACTCTCTTCTAGTTCATAGCAATAAAAATTTAGTGATTTTAAATTTTACTAGTTCCTCATTTTCATTT 5700
     ||||||||||||||||||||||||||||||||||||||||||||||||||||||||||||||||||||||||||||||||||||||||||||||||||||||||||||||||||||||||||||||||||||||||||||||||||||
5545 GCAATAACATTGAATTGAGCAATGGTGTTTTTGTTCTTTATTTAAACACATGTGATGTATTAGAACGAAAAAATATATATATACTCTCTTCTAGTTCATAGCAATAAAAATTTAGTGATTTTAAATTTTACTAGTTCCTCATTTTCATTT 5694
          *         *         *         *         *         *         *         *         *         *         *         *         *         *         *

              *         *         *         *         *         *         *         *         *         *         *         *         *         *         *
5701 TAAATTTTTTATAACAATTTAATCTAAATATTTGCTCAAATATATAATTTAATATGATACATAAATAAAACAAGATCGATCCAAAATACACGAGAGTATAGTTAGAAAACGACGGCAAAAACTTTTGTATGTAGAGGTACTTTCATATAA 5850
     ||||||||||||||||||||||||||||||||||||||||||||||||||||||||||||||||||||||||||||||||||||||||||||||||||||||||||||||||||||||||||||||||||||||||||||||||||||
5695 TAAATTTTTTATAACAATTTAATCTAAATATTTGCTCAAATATATAATTTAATATGATACATAAATAAAACAAGATCGATCCAAAATACACGAGAGTATAGTTAGAAAACGACGGCAAAAACTTTTGTATGTAGAGGTACTTTCATATAA 5844
          *         *         *         *         *         *         *         *         *         *         *         *         *         *         *

              *         *         *         *         *         *         *         *         *         *         *         *         *         *         *
5851 ATATTTAAGTTCACAGTTTTAAGAAAACTTTCATAAATATTTTTAAACTATTATTAAAATAAAATTTATTAAAAATTTAAAATAAAAATGGAGACTAATTAGTACGTGGTGATACAATTCCATCTTAAATGGCTATGACACCATTTCATG 6000
     ||||||||||||||||||||||||||||||||||||||||||||||||||||||||||||||||||||||||||||||||||||||||||||||||||||||||||||||||||||||||||||||||||||||||||||||||||||
5845 ATATTTAAGTTCACAGTTTTAAGAAAACTTTCATAAATATTTTTAAACTATTATTAAAATAAAATTTATTAAAAATTTAAAATAAAAATGGAGACTAATTAGTACGTGGTGATACAATTCCATCTTAAATGGCTATGACACCATTTCATG 5994
          *         *         *         *         *         *         *         *         *         *         *         *         *         *         *

              *         *         *         *         *         *         *         *         *         *         *         *         *         *         *
6001 AGTTAATTAAAGAAAATTTTGAAACATCTAATAACTTACTACTTTTGAAAGTAGTAAGGATACCTTTTAAACAAATGATAAGTAATTTTATAATTTAATCAAACAAGCAAAAGGGTATAAAATGGGATTGTGGAGAAAGGCAAGAAATTA 6150
     ||||||||||||||||||||||||||||||||||||||||||||||||||||||||||||||||||||||||||||||||||||||||||||||||||||||||||||||||||||||||||||||||||||||||||||||||||||
5995 AGTTAATTAAAGAAAATTTTGAAACATCTAATAACTTACTACTTTTGAAAGTAGTAAGGATACCTTTTAAACAAATGATAAGTAATTTTATAATTTAATCAAACAAGCAAAAGGGTATAAAATGGGATTGTGGAGAAAGGCAAGAAATTA 6144
          *         *         *         *         *         *         *         *         *         *         *         *         *         *         *

              *         *         *         *         *         *         *         *         *         *         *         *         *         *         *
6151 ATTAGAAAGAAAAAGAATAGGTCCAACCACCAAGTCTTTGCATGTGCGGCGCCTTGCTTCCATTCTAAACTTTTCACTTCACCCCACGAGCATACTGCCGCGTGTATTACTATTAAATTCGCAGATACACGATTTGAAACCAAAGCTGGT 6300
     ||||||||||||||||||||||||||||||||||||||||||||||||||||||||||||||||||||||||||||||||||||||||||||||||||||||||||||||||||||||||||||||||||||||||||||||||||||
6145 ATTAGAAAGAAAAAGAATAGGTCCAACCACCAAGTCTTTGCATGTGCGGCGCCTTGCTTCCATTCTAAACTTTTCACTTCACCCCACGAGCATACTGCCGCGTGTATTACTATTAAATTCGCAGATACACGATTTGAAACCAAAGCTGGT 6294
          *         *         *         *         *         *         *         *         *         *         *         *         *         *         *

              *         *         *         *         *         *         *         *         *         *         *         *         *         *         *
6301 GGTGGATATGCATATGAGTGCGCGTGGAGAGTTACACTTATAGAGACGAACTTTTGTTGAAGAAGGTATTATTAAATGATGGGGTTTGACGCTCACACCCCTGTTTCCTCAACACTCTCTACCCTAAACTACTAACTTACACTTCACTTC 6450
     ||||||||||||||||||||||||||||||||||||||||||||||||||||||||||||||||||||||||||||||||||||||||||||||||||||||||||||||||||||||||||||||||||||||||||||||||||||
6295 GGTGGATATGCATATGAGTGCGCGTGGAGAGTTACACTTATAGAGACGAACTTTTGTTGAAGAAGGTATTATTAAATGATGGGGTTTGACGCTCACACCCCTGTTTCCTCAACACTCTCTACCCTAAACTACTAACTTACACTTCACTTC 6444
          *         *         *         *         *         *         *         *         *         *         *         *         *         *         *

              *         *         *         *         *         *         *         *         *         *         *         *         *         *         *
6451 ACATCATCTCAATCCTAATTTCAGTTACTGACCTGATTCATCCTATCTTATTTTGTTCAACACCTAACTAGACATCCAATAAAAGACCTCACAAACATCTCAAATGTATGTTAATTCACTTGTTTCGACTCTGGCTTACTAGACTATGAT 6600
     ||||||||||||||||||||||||||||||||||||||||||||||||||||||||||||||||||||||||||||||||||||||||||||||||||||||||||||||||||||||||||||||||||||||||||||||||||||
6445 ACATCATCTCAATCCTAATTTCAGTTACTGACCTGATTCATCCTATCTTATTTTGTTCAACACCTAACTAGACATCCAATAAAAGACCTCACAAACATCTCAAATGTATGTTAATTCACTTGTTTCGACTCTGGCTTACTAGACTATGAT 6594
```

```
6601 GCACTTTGTTGATGAAGAGTTTGCAAATTGGAGACTTATGAGCTAACATAAGTTTGATAAGTCATTTTAATATGAGGAGTTAACAAAGAAAAAAAAAAAAGTCTTTAACAAGGATTGTATAACGGTTATATATGGAACTTTTTTTTTCCTT 6750
     ||||||||||||||||||||||||||||||||||||||||||||||||||||||||||||||||||||||||||||||||||||||||||||||||||||||||||||||||||||||||||||||||||||||||||||||||||||
6595 GCACTTTGTTGATGAAGAGTTTGCAAATTGGAGACTTATGAGCTAACATAAGTTTGATAAGTCATTTTAATATGAGGAGTTAACAAAGAAAAAAAAAAAAGTCTTTAACAAGGATTGTATAACGGTTATATATGGAACTTTTTTTTTCCTT 6744

6751 CGCCAGTTGGTTATTGTTTTCGTATACCTAAGTCATTGTGCAAGTTAGGTGAGTTCTCCTTCCCAAATTTGAGGTTTTTTTCTTTAAACCAAATTAAGGAGAAAAAATTAAAGTTGTATTTAGTAGGTAATTTAAATTATGTTTTATATT 6900
     ||||||||||||||||||||||||||||||||||||||||||||||||||||||||||||||||||||||||||||||||||||||||||||||||||||||||||||||||||||||||||||||||||||||||||||||||||||
6745 CGCCAGTTGGTTATTGTTTTCGTATACCTAAGTCATTGTGCAAGTTAGGTGAGTTCTCCTTCCCAAATTTGAGGTTTTTTTCTTTAAACCAAATTAAGGAGAAAAAATTAAAGTTGTATTTAGTAGGTAATTTAAATTATGTTTTATATT 6894

6901 TTTGAGATTCATGGGTTTGTCCAATAATCGAAATTATATTTTTGTTTTCAACTGTCGTAATCTCTCTAACATTTTAGTTCTTTTCAATGTATTTTTATAAGCATTTTATAATGGAATGGTTTTTTTTTCTAAATGTAATTAAGTTTTTA 7050
     ||||||||||||||||||||||||||||||||||||||||||||||||||||||||||||||||||||||||||||||||||||||||||||||||||||||||||||||||||||||||||||||||||||||||||||||||||||
6895 TTTGAGATTCATGGGTTTGTCCAATAATCGAAATTATATTTTTGTTTTCAACTGTCGTAATCTCTCTAACATTTTAGTTCTTTTCAATGTATTTTTATAAGCATTTTATAATGGAATGGTTTTTTTTTCTAAATGTAATTAAGTTTTTA 7044

7051 CCTCTAAAGAACATTGTGTAATTAGCTCAAATTTTTATGTGTCTGGAAAGTCAAAATACAAAACTTTATGTTTTAGTTTCTTTATAAAATATAGATATATCCAACTCACCATATAAAATTTAAAACTTAATTAATTTGTTAATGACTTGT 7200
     ||||||||||||||||||||||||||||||||||||||||||||||||||||||||||||||||||||||||||||||||||||||||||||||||||||||||||||||||||||||||||||||||||||||||||||||||||||
7045 CCTCTAAAGAACATTGTGTAATTAGCTCAAATTTTTATGTGTCTGGAAAGTCAAAATACAAAACTTTATGTTTTAGTTTCTTTATAAAATATAGATATATCCAACTCACCATATAAAATTTAAAACTTAATTAATTTGTTAATGACTTGT 7194

7201 AACAGTGTGTTAAATGAGTTAAACTTGATTTAATATTACATCTTTTTATTGTGGATTAGTCTAATTTAAATTAGGCATCACTTATCATTATTTCAAAATGAATTATATGGTACATTCAGTTACCTACTTTGATTGGGGTTTTTTTTTTTT 7350
     ||||||||||||||||||||||||||||||||||||||||||||||||||||||||||||||||||||||||||||||||||||||||||||||||||||||||||||||||||||||||||||||||||||||||||||||||||||
7195 AACAGTGTGTTAAATGAGTTAAACTTGATTTAATATTACATCTTTTTATTGTGGATTAGTCTAATTTAAATTAGGCATCACTTATCATTATTTCAAAATGAATTATATGGTACATTCAGTTACCTACTTTGATTGGGGTTTTTTTTTTTT 7344

7351 TTTTCTTGAACAAAATTAAAAAATAAGAAAAACATTAAAAACTCATATATATATTTTAATGCAATTTTAAAAGTGAATCAAGTGGTTTAATTTTAACATACTATTATATGTGTTATATATATACTTGAGAATCTCACATGAAAAACACTT 7500
     ||||||||||||||||||||||||||||||||||||||||||||||||||||||||||||||||||||||||||||||||||||||||||||||||||||||||||||||||||||||||||||||||||||||||||||||||||||
7345 TTTTCTTGAACAAAATTAAAAAATAAGAAAAACATTAAAAACTCATATATATATTTTAATGCAATTTTAAAAGTGAATCAAGTGGTTTAATTTTAACATACTATTATATGTGTTATATATATACTTGAGAATCTCACATGAAAAACACTT 7494

7501 GAAAAAAAGATTTAATATTGATGATCTAATGGCATACTTCTTAAATTGACATCACCAAGCTGTTTTCTAAACTGTAAATCAAAAGTGTTGCATAAAAAACTACCTCTAAACTATAAATTTTGTTGTAATCATACTTTCCAATTCTCAATT 7650
     ||||||||||||||||||||||||||||||||||||||||||||||||||||||||||||||||||||||||||||||||||||||||||||||||||||||||||||||||||||||||||||||||||||||||||||||||||||
7495 GAAAAAAAGATTTAATATTGATGATCTAATGGCATACTTCTTAAATTGACATCACCAAGCTGTTTTCTAAACTGTAAATCAAAAGTGTTGCATAAAAAACTACCTCTAAACTATAAATTTTGTTGTAATCATACTTTCCAATTCTCAATT 7644

7651 ACACCGATCACCTTCTTTATTTTTTTTTTCAAAAAAATAATGTTGAAAGTATTCATAAATTATAAATTTTGTTCCATATAAGTTTCAACAAAGTACATAATTATAATAAAACCTTTATTGTTCTAGTCGATTTAGGTTAAACATCTATT 7800
     ||||||||||||||||||||||||||||||||||||||||||||||||||||||||||||||||||||||||||||||||||||||||||||||||||||||||||||||||||||||||||||||||||||||||||||||||||||
7645 ACACCGATCACCTTCTTTATTTTTTTTTTCAAAAAAATAATGTTGAAAGTATTCATAAATTATAAATTTTGTTCCATATAAGTTTCAACAAAGTACATAATTATAATAAAACCTTTATTGTTCTAGTCGATTTAGGTTAAACATCTATT 7794

7801 AAACATTATTTTAAGTTTAAAAAAAAAAAAACTAAATAGATCACAAATCATATTTAAATGTTTAATGACTTAATTTGAAATTTAACCAACTAAAAATACGTACTTTAAAAAAAGCAACATTTGAAGTTGATGATATTACTACCATAGGGA 7950
     ||||||||||||||||||||||||||||||||||||||||||||||||||||||||||||||||||||||||||||||||||||||||||||||||||||||||||||||||||||||||||||||||||||||||||||||||||||
7795 AAACATTATTTTAAGTTTAAAAAAAAAAAAACTAAATAGATCACAAATCATATTTAAATGTTTAATGACTTAATTTGAAATTTAACCAACTAAAAATACGTACTTTAAAAAAAGCAACATTTGAAGTTGATGATATTACTACCATAGGGA 7944

7951 TAAGTAGAGAAAATATAGTATTGCATAGAGATAAGTGGTCGATTGTTCGTTATCTCTTATTTGAGAAGAATATAGATAGATAATGTAATTGGAATCAGCAATTAAGCATAAAAAAATGTGTTGATCTAGTAATTACCTCCATTATTAAAC 8100
     ||||||||||||||||||||||||||||||||||||||||||||||||||||||||||||||||||||||||||||||||||||||||||||||||||||||||||||||||||||||||||||||||||||||||||||||||||||
7945 TAAGTAGAGAAAATATAGTATTGCATAGAGATAAGTGGTCGATTGTTCGTTATCTCTTATTTGAGAAGAATATAGATAGATAATGTAATTGGAATCAGCAATTAAGCATAAAAAAATGTGTTGATCTAGTAATTACCTCCATTATTAAAC 8094

8101 TTGAGTCTTTGATGTGTTGGATATATATTGTTAATTAAACTCCCGAAGGTCATTTCTTTAAATGAGATTGGAATTTTCATGGAAGATATTTCTTTTTTGTTGAGAATAATGTTACTAATGTTGTTGATTTTTGTTATATCTCTGGAAAGA 8250
     ||||||||||||||||||||||||||||||||||||||||||||||||||||||||||||||||||||||||||||||||||||||||||||||||||||||||||||||||||||||||||||||||||||||||||||||||||||
8095 TTGAGTCTTTGATGTGTTGGATATATATTGTTAATTAAACTCCCGAAGGTCATTTCTTTAAATGAGATTGGAATTTTCATGGAAGATATTTCTTTTTTGTTGAGAATAATGTTACTAATGTTGTTGATTTTTGTTATATCTCTGGAAAGA 8244

8251 TGAACAAAGTTATTCATTCTACTGTAGACTTTAGCCTTCCTTTTAACGAAGTGTTCTTTAATCTGAGAAGATGTATCCGAGATGTGTTCAGTTCCTCTTGATTTGATTGGGAGCTTGAGAGAGTTTAATTTGGTTATGGTGTTGCTCTTG 8400
     ||||||||||||||||||||||||||||||||||||||||||||||||||||||||||||||||||||||||||||||||||||||||||||||||||||||||||||||||||||||||||||||||||||||||||||||||||||
8245 TGAACAAAGTTATTCATTCTACTGTAGACTTTAGCCTTCCTTTTAACGAAGTGTTCTTTAATCTGAGAAGATGTATCCGAGATGTGTTCAGTTCCTCTTGATTTGATTGGGAGCTTGAGAGAGTTTAATTTGGTTATGGTGTTGCTCTTG 8394

8401 ATTGCCTGTCTTTTGCCTAAAAAAACAAAACAAATAATGTAAGTGAAACCAAACATATAAATCTCTTATCACTTACCTTATCATTTTCACTCTCTCTCTCTCTCTCTCTTCCAATAATATAAAGAGAGGAAGTCCTCTCATTGGCCATTT 8550
     ||||||||||||||||||||||||||||||||||||||||||||||||||||||||||||||||||||||||||||||||||||||||||||||||||||||||||||||||||||||||||||||||||||||||||||||||||||
8395 ATTGCCTGTCTTTTGCCTAAAAAAACAAAACAAATAATGTAAGTGAAACCAAACATATAAATCTCTTATCACTTACCTTATCATTTTCACTCTCTCTCTCTCTCTCTCTTCCAATAATATAAAGAGAGGAAGTCCTCTCATTGGCCATTT 8544

8551 TTGAAATTCACACACACACAAAAGAAAAAACAATGCCAACCAAACTTGGAAGAAACTATTTGAACCTTTGTTTTACAAAGATCAAAAATCCTCTCTCCACCGCTCAATCCTCTCCCCCCATCACCCACACCGCAGATCGCCGCCGTCAAA 8700
     ||||||||||||||||||||||||||||||||||||||||||||||||||||||||||||||||||||||||||||||||||||||||||||||||||||||||||||||||||||||||||||||||||||||||||||||||||||
8545 TTGAAATTCACACACACACAAAAGAAAAAACAATGCCAACCAAACTTGGAAGAAACTATTTGAACCTTTGTTTTACAAAGATCAAAAATCCTCTCTCCACCGCTCAATCCTCTCCCCCCATCACCCACACCGCAGATCGCCGCCGTCAAA 8694

8701 CCACCCGTTCCTTCTCCTCCACTGCTGCCGCCTTCATCACAAACTACAACTCTCTCTACGAGATCACCACCACCACCACCACAAACTCCGACTCCAATTCCCCCTCCACACCCCTTTTCGGGCTCACCAATGACATTGGTGTCGCCGATC 8850
     ||||||||||||||||||||||||||||||||||||||||||||||||||||||||||||||||||||||||||||||||||||||||||||||||||||||||||||||||||||||||||||||||||||||||||||||||||||
8695 CCACCCGTTCCTTCTCCTCCACTGCTGCCGCCTTCATCACAAACTACAACTCTCTCTACGAGATCACCACCACCACCACCACAAACTCCGACTCCAATTCCCCCTCCACACCCCTTTTCGGGCTCACCAATGACATTGGTGTCGCCGATC 8844

8851 CGGACGCCTATGTTGCAGTCGATTTCATTACCGCCTTCACCTCCCATCGTTTCTTCTTCTCCTCTCCTGGCAGCTCCAACTCCATCATAGAATCCACCACTACCACTACTACCGAATCCACAACCACCATGTCACTGTCGTCCGAGTACT 9000
     ||||||||||||||||||||||||||||||||||||||||||||||||||||||||||||||||||||||||||||||||||||||||||||||||||||||||||||||||||||||||||||||||||||||||||||||||||||
8845 CGGACGCCTATGTTGCAGTCGATTTCATTACCGCCTTCACCTCCCATCGTTTCTTCTTCTCCTCTCCTGGCAGCTCCAACTCCATCATAGAATCCACCACTACCACTACTACCGAATCCACAACCACCATGTCACTGTCGTCCGAGTACT 8994

9001 CCGCCAGGTACGAAGGCAACGACGACGATTTGATGATCTTCAACAATAGCCATGTGATCCCCACGTACTCACCAGACCCATACATGGACTTCCGACGGTCAATGCAAGAGATGATGGAAGCGCGTGAGAAAATGACGACGGCGGTGGCAA 9150
     ||||||||||||||||||||||||||||||||||||||||||||||||||||||||||||||||||||||||||||||||||||||||||||||||||||||||||||||||||||||||||||||||||||||||||||||||||||
8995 CCGCCAGGTACGAAGGCAACGACGACGATTTGATGATCTTCAACAATAGCCATGTGATCCCCACGTACTCACCAGACCCATACATGGACTTCCGACGGTCAATGCAAGAGATGATGGAAGCGCGTGAGAAAATGACGACGGCGGTGGCAA 9144

9151 CGACAACAACGATGAAAAAGTCAAGTTGGGAATTCTTGCATGAACTTCTTTTATGCTATCTAGCACTCAACCCTAAAACCACTCATAAACATATCCTCAAGGCTTTCGCGGACATTGCGACAGTAATAAAACCGCCATTGGCGATGAAGG 9300
     ||||||||||||||||||||||||||||||||||||||||||||||||||||||||||||||||||||||||||||||||||||||||||||||||||||||||||||||||||||||||||||||||||||||||||||||||||||
9145 CGACAACAACGATGAAAAAGTCAAGTTGGGAATTCTTGCATGAACTTCTTTTATGCTATCTAGCACTCAACCCTAAAACCACTCATAAACATATCCTCAAGGCTTTCGCGGACATTGCGACAGTAATAAAACCGCCATTGGCGATGAAGG 9294

9301 AAACGGAGGAAGAGGAGAATGTGGATCGGGAAAAAGGTGAGTCAATGGTGGACGACAGAGGGGCGGGCGGCGGTGGCTGTGAGTGTGAGATGTCCGGCCAACAAAACGATAGAGATTGATGCATACATATAATAATGATTAATTATTAAA 9450
     ||||||||||||||||||||||||||||||||||||||||||||||||||||||||||||||||||||||||||||||||||||||||||||||||||||||||||||||||||||||||||||||||||||||||||||||||||||
9295 AAACGGAGGAAGAGGAGAATGTGGATCGGGAAAAAGGTGAGTCAATGGTGGACGACAGAGGGGCGGGCGGCGGTGGCTGTGAGTGTGAGATGTCCGGCCAACAAAACGATAGAGATTGATGCATACATATAATAATGATTAATTATTAAA 9444

9451 TGTGGGGTTGCAGGAAAATATTGATTAGATTAATTATAAGTTTTTGATATGATTTTGAGTTTTATGAATATCAATATATAATATGATTATAATATAATGTGGTGTAGGGGGGAGTGAAGAAGAAGAAGAAGAAGAAGAAGAAGAGGTGGG 9600
     ||||||||||||||||||||||||||||||||||||||||||||||||||||||||||||||||||||||||||||||||||||||||||||||||||||||||||||||||||||||||||||||||||||||||||||||||||||
9445 TGTGGGGTTGCAGGAAAATATTGATTAGATTAATTATAAGTTTTTGATATGATTTTGAGTTTTATGAATATCAATATATAATATGATTATAATATAATGTGGTGTAGGGGGGAGTGAAGAAGAAGAAGAAGAAGAAGAAGAAGAGGTGGG 9594

9601 TCCCACATGACTTTGGTTTTCGTCTTTCTCACCTTAATTAATTATTACATCAAAACCCTTTTTCTCTTTACTTTTTGTTTCCATAACATTTTTTGCTCACATCTTCTTTTACATTTTGTATATCTTTTTGTTGCCACAGGATCAAACAGT 9750
     ||||||||||||||||||||||||||||||||||||||||||||||||||||||||||||||||||||||||||||||||||||||||||||||||||||||||||||||||||||||||||||||||||||||||||||||||||||
9595 TCCCACATGACTTTGGTTTTCGTCTTTCTCACCTTAATTAATTATTACATCAAAACCCTTTTTCTCTTTACTTTTTGTTTCCATAACATTTTTTGCTCACATCTTCTTTTACATTTTGTATATCTTTTTGTTGCCACAGGATCAAACAGT 9744

9751 GTTATTATTATCTAATTTCAAGCATGAGCTTATCACAATTTTAATCTAACATGGCCTTTTACATGTTACATTAATTCCATTTTTCATTCATTTACATTCATAAACTATAAAGGTCTACCATCTTCTTTCTATAAAATCTTTGATAACTTT 9900
     ||||||||||||||||||||||||||||||||||||||||||||||||||||||||||||||||||||||||||||||||||||||||||||||||||||||||||||||||||||||||||||||||||||||||||||||||||||
9745 GTTATTATTATCTAATTTCAAGCATGAGCTTATCACAATTTTAATCTAACATGGCCTTTTACATGTTACATTAATTCCATTTTTCATTCATTTACATTCATAAACTATAAAGGTCTACCATCTTCTTTCTATAAAATCTTTGATAACTTT 9894

9901 TCAAATAGGTTAAAGACTACTTGTAATTTATCGTAGTCTTTAAATAATTAGTTCACAAACGTCATAGATGAGAAGCTTAGACAATTGAAAAACTAATAGGGACATGTAAAAGTTTAAATATTAATTGTGTTCATTTATCTTAAATTAAAT 10050
```

```
9895 TCAAATAGGTTAAAGACTACTTGTAATTTATCGTAGTCTTTAAATAATTAGTTCACAAACGTCATAGATGAGAAGCTTAGACAATTGAAAACTAATAGGGACATGTAAAAGTTTAAATATTAATTGTGTTCATTTATCTTAAATTAAAT 10044

10051 CCTGATTTTAAAAGTGAATAAAATAAAAATGAAATGGTGAAGAGAGTACATTAAGTTATCCACTTTAAGTTTAATATATAATATAACATGAGACAAAATTTAATGGAATAATTATGTACTAGTCAAAGTTGTGGAGTGTATTGATAATT 10200
10045 CCTGATTTTAAAAGTGAATAAAATAAAAATGAAATGGTGAAGAGAGTACATTAAGTTATCCACTTTAAGTTTAATATATAATATAACATGAGACAAAATTTAATGGAATAATTATGTACTAGTCAAAGTTGTGGAGTGTATTGATAATT 10194

10201 TAACATAAAGGCTAAAGACAGATTTCTTATTCAATTGTTATGGATCCTTCTGATTGAGGCTTTGTTGGTTTTTTCTGTAGTTTTTTTAACACCTTTTATCTAGATGGTATAATTTGACATGACCTAGTAATTAAATGGTGGAAAAAAGA 10350
10195 TAACATAAAGGCTAAAGACAGATTTCTTATTCAATTGTTATGGATCCTTCTGATTGAGGCTTTGTTGGTTTTTTCTGTAGTTTTTTTAACACCTTTTATCTAGATGGTATAATTTGACATGACCTAGTAATTAAATGGTGGAAAAAAGA 10344

10351 AAGAATAGATCAGACAAAAAAAAAAAAGTTAAAAGACGACAAATCGATGATGTATTAAATATATAGAGGAATATTGAGATGCATCTCAAAATATACTTTATCTACATACATCATATAATTCTCGTTATATACCAAAGTTTAAAACATCAA 10500
10345 AAGAATAGATCAGACAAAAAAAAAAAAGTTAAAAGACGACAAATCGACGATGTATTAAATATATAGAGGAATATTGAGATGCATCTCAAAATATACTTTATCTACATACATCATATAATTCTCGTTATATACCAAAGTTTAAAACATCAA 10494

10501 TGTCAATGAATATGTCGATGTCTTAATTTTAGGAAAAATTCAATGGAAATATCGATAAGAAATGTTTTTGAAAATATTTTAAAATTAAAAATTTTTAAAATAAATTTAAAGTGATAAATAATATTTTGCCACGTGTTTAATTATAGTCGA 10650
10495 TGTCAATGAATATGTCGATGTCTTAATTTTAGGAAAAATTCAATGGAAATATCGATAAGAAATGTTTTTGAAAATATTTTAAAATTAAAAATTTTTAAAATAAATTTAAAGTGATAAATAATATTTTGCCACGTGTTTAATTATAGTCGA 10644

10651 GGTTTATTTTTCATGTTTTGTGGATTCTTCCACTGTGTAATATGCAAATATTGATTCAATGTCATATGTTAATTAAGCTTTGTACATGTAATCTCAAAGTAATAATTCAAAGAATGTGATTGAATTATTCTTCAAATAATAAAAATAAAT 10800
10645 GGTTTATTTTTCATGTTTTGTGGATTCTTCCACTGTGTAATATGCAAATATTGATTCAATGTCATATGTTAATTAAGCTTTGTACATGTAATCTCAAAGTAATAATTCAAAGAATGTGATTGAATTATTCTTCAAATAATAAAAATAAAT 10794

10801 AATAATGGATGAATTTACAACCACGAAGAAGTTTCCAAAATTGTCAGTGAAAATTTGCCTACGAATTGTATGTCACGTCAACGTACTTTTAAAAATAAAATGAGATTCATTGACATAAATACAAAGTCAATAAATATTATTTATGAATAT 10950
10795 AATAATGGATGAATTTACAACCACGAAGAAGTTTCCAAAATTGTCAGTGAAAATTTGCCTACGAATTGTATGTCACGTCAACGTACTTTTAAAAATAAAATGAGATTCATTGACATAAATACAAAGTCAATAAATATTATTTATGAATAT 10944

10951 AAATTTATAGTGTGAATAAAAAAAATATGATAGTTCTTTCGTGGTACTAGAAATATTCTTAAGATAATACTCACATATAGAGAAGGCATTTGTAATTTTGATCCAAATTTGGTCTGTATGGTGTGGTTAAAAGTAGAATTTTGTTCATAT 11100
10945 AAATTTATAGTGTGAATAAAAAAAATATGATAGTTCTTTCGTGGTACTAGAAATATTCTTAAGATAATACTCACATATAGAGAAGGCATTTGTAATTTTGATCCAAATTTGGTCTGTATGGTGTGGTTAAAAGTAGAATTTTGTTCATAT 11094

11101 GCTTTGATAAAACCATCCTAAATAGTTTTTCTTATAGAGACTAAATCAAACCATAATTGACTAAATCTTAATTATAAATCATAATAACTAAACTCTAAAATTCTCCAAACTAGCTAGATATTATGGTTTAATAAACATAGATATATATAT 11250
11095 GCTTTGATAAAACCATCCTAAATAGTTTTTCTTATAGAGACTAAATCAAACCATAATTGACTAAATCTTAATTATAAATCATAATAACTAAACTCTAAAATTCTCCAAACTAGCTAGATATTATGGTTTAATAAACATAGATATATATAT 11244

11251 GTATATATATATATATGTATGCATTGAAAATTTTGATAATAAAGACAGTATATAAAGATGAAAAATAGAAATTGGCAGTTAGGAAGCAAACGTAAAGATAAAAATGTAAGAGCCCCAAAACATTTCTGTGAAATTTGATGTGAAGCAACA 11400
11245 GTATATATATATATATGTATGCATTGAAAATTTTGATAATAAAGACAGTATATAAAGATGAAAAATAGAAATTGGCAGTTAGGAAGCAAACGTAAAGATAAAAATGTAAGAGCCCCAAAACATTTCTGTGAAATTTGATGTGAAGCAACA 11394

11401 CATGAAAAGCACTCACAGTACTGTCATATAGATCAACATAAAGCAAATCATAAGAAGAGATAATGCCTTCGTCTCTTCTTCTTTTTTCTCTCTCACAAATTCTCTCACTTTTCTCCTCCTTTCTCATAACCAAACAAAACCCAATTTCCA 11550
11395 CATGAAAAGCACTCACAGTACTGTCATATAGATCAACATAAAGCAAATCATAAGAAGAGATAATGCCTTCGTCTCTTCTTCTTTTTTCTCTCTCACAAATTCTCTCACTTTTCTCCTCCTTTCTCATAACCAAACAAAACCCAATTTCCA 11544

11551 TAATATTTTTTTTTATAAATAAAAAATAAACCACATGGGGTACACACAAAAAACCATATCTCCTTCTGCAAAACACATGCTGATCTTATAAGAAAGAAACCCTAATTTGAATGGGCCACATGGAACCATATTCCAAAACAGCCCTTCTCT 11700
11545 TAATATTTTTTTTTATAAATAAAAAATAAACCACATGGGGTACACACAAAAAACCATATCTCCTTCTGCAAAACACATGCTGATCTTATAAGAAAGAAACCCTAATTTGAATGGGCCACATGGAACCATATTCCAAAACAGCCCTTCTCT 11694

11701 CACTCCCATGAAAAAAGACAGTTAAAAACAATAATGATAAGTTACTGTCATTAAAGGACAATTTAGAGAAAACAAATAATAATAATAACACAACAATATTGTAATATTTCTTTGAGATTGGTTGTCCAATGTTGTGGAGACAGATGATG 11850
11695 CACTCCCATGAAAAAAGACAGTTAAAAACAATAATGATAAGTTACTGTCATTAAAGGACAATTTAGAGAAAACAAATAATAATAATAACACAACAATATTGTAATATTTCTTTGAGATTGGTTGTCCAATGTTGTGGAGACAGATGATG 11844

11851 GTTGTACTTAATAACATTACATTTGGAGGCCAAATGGGAATCTTTGGATTGTAAATTTTTTTAATTTATCAATGCTGCCAAATTGGGGGAATCTTTATTATTTTGTCTTCTCTTTTCTTTTTATTTATTTATTTGAGTTTTATTTGTTCT 12000
11845 GTTGTACTTAATAACATTACATTTGGAGGCCAAATGGGAATCTTTGGATTGTAAATTTTTTTAATTTATCAATGCTGCCAAATTGGGGGAATCTTTATTATTTTGTCTTCTCTTTTCTTTTTATTTATTTATTTGAGTTTTATTTGTTCT 11994

12001 AAGTTTGAATGGGAGGGAAAACTTCAAAACTAGAAGTGGGTGGGTGGGTGGGTGGATTCATATGCACTCATGAGATTCAGTGAATTTTCCTTTAACCAAGTGGAATACTTTAAAGCTTTATGTTGAGTACTTCTTACTTAAATTCTATTT 12150
11995 AAGTTTGAATGGGAGGGAAAACTTCAAAACTAGAAGTGGGTGGGTGGGTGGGTGGATTCATATGCACTCATGAGATTCAGTGAATTTTCCTTTAACCAAGTGGAATACTTTAAAGCTTTATGTTGAGTACTTCTTACTTAAATTCTATTT 12144

12151 TCCACTCTTTTTCTTTTTCATGTGGTGGATTTCTTGATCATAGTGTAATTTGAGTAACCTCAATTTACAATTTTGGGGGAAACTTAATTGAGGGCTACTAATTTTCTGAAACCGTGTTTGTGTTAGTTATCATAGTTAATGGTTGAATTA 12300
12145 TCCACTCTTTTTCTTTTTCATGTGGTGGATTTCTTGATCATAGTGTAATTTGAGTAACCTCAATTTACAATTTTGGGGGAAACTTAATTGAGGGCTACTAATTTTCTGAAACCGTGTTTGTGTTAGTTATCATAGTTAATGGTTGAATTA 12294

12301 TTATAGTTGGTGTTTGAAACTATTTTAGTTTAGGTATAAAATAGTTAATAGAATAACAAAAGAAAAAGGAAAAAAAAAAATTAGAAATAGTAAATACTATGACAAAAAGAAATTTCAAATGGTTGAAAACACAAACTATTATATTTGGAGG 12450
12295 TTATAGTTGGTGTTTGAAACTATTTTAGTTTAGGTATAAAATAGTTAATAGAATAACAAAAGAAAAAGGAAAAAAAAAAATTAGAAATAGTAAATACTATGACAAAAAGAAATTTCAAATGGTTGAAAACACAAACTATTATATTTGGAGG 12444

12451 TTCAAATACCGAGTAAGCACTTCACTGACTTGAAGTTGGAACCCTATGGTTTTAAAAGTTAGACTATGATTAACGGTTGTTATTACTACTATTATCATGAGTTTATTGGATTGTTATTCTTCCCGTTCAATTATATGTATCTAATAGGTT 12600
12445 TTCAAATACCGAGTAAGCACTTCACTGACTTGAAGTTGGAACCCTATGGTTTTAAAAGTTAGACTATGATTAACGGTTGTTATTACTACTATTATCATGAGTTTATTGGATTGTTATTCTTCCCGTTCAATTATATGTATCTAATAGGTT 12594

12601 CTAAAACGTTGAATTTTGTTGAATAAACCCTCAACTTTTTTTTTATGTCTAATAAGTCTTCACATATTCAACATTTTTACTAAAATTAATTGATCTATTTAATATAAATTAGAATTGTATGTGTAATAAAATCCACAATTTTCGACTTGG 12750
12595 CTAAAACGTTGAATTTTGTTGAATAAACCCTCAACTTTTTTTTTATGTCTAATAAGTCTTCACATATTCAACATTTTTACTAAAATTAATTGATCTATTTAATATAAATTAGAATTGTATGTGTAATAAAATCCACAATTTTCGACTTGG 12744

12751 ACCTAGTAGATCAACAAATATGAAAAAATTGAAACAATACTTCATAAAAATCTCAAGCTCGAGGGGCAAAAGGAGGGCACAAATCAAGGTTAATTTCATCCAAAAACCAACCACGGAACACAAGAGTCTCAAGCCTTTCCAAAATCAGTA 12900
12745 ACCTAGTAGATCAACAAATATGAAAAAATTGAAACAATACTTCATAAAAATCTCAAGCTCGAGGGGCAAAAGGAGGGCACAAATCAAGGTTAATTTCATCCAAAAACCAACCACGGAACACAAGAGTCTCAAGCCTTTCCAAAATCAGTA 12894

12901 AGACTTAAACCCAACTGCATTTGAGAGCTAGAAAGCGAGCAATCCGATTTTCCCACACAAGGACAAAATGAGAAACATTTCCCTAACCTCCTCACCATCACAGTAATCTCATCAACTACATTTTTTACCTTGAGACTAAATTTGTACTAT 13050
12895 AGACTTAAACCCAACTGCATTTGAGAGCTAGAAAGCGAGCAATCCGATTTTCCCACACAAGGACAAAATGAGAAACATTTCCCTAACCTCCTCACCATCACAGTAATCTCATCAACTACATTTTTTACCTTGAGACTAAATTTGTACTAT 13044

13051 TGAAAAATTTAGAGACTAAATAGATAAAAAATTTGAAAATTAAGGCAATAAATCTGTAATTTTACCAAAAGTTTATCTTATCAACCTAGCAGGCACACACTAATTACACAGTATCTCCTTTCGCTCATAATATTGTTCAACAGCACCTATA 13200
13045 TGAAAAATTTAGAGACTAAATAGATAAAAAATTTGAAATTTAAGGCAATAAATCTGTAATTTTACCAAAAGTTTATCTTATCAACCTAGCAGGCACACACTAATTACACAGTATCTCCTTTCGCTCATAATATTGTTCAACAGCACCTATA 13194

13201 GAAAAGAAAATGTAGGAAATTGCACAATCTTCAAATTTACCAACATTCCAATGATCTAAAATTCATCTACCTATTTAACCCAAATTTCTCATCAATATGAGAAATTTGGCTTAGCACAAAGAAGAAGAGAACCAATAACTGTGATCTATT 13350
13195 GAAAAGAAAATGTAGGAAATTGCACAATCTTCAAATTTACCAACATTCCAATGATCTAAAATTCATCTACCTATTTAACCCAAATTTCTCATCAATATGAGAAATTTGGCTTAGCACAAAGAAGAAGAGAACCAATAACTGTGATCTATT 13344
```

```
               *         *         *         *         *         *         *         *         *         *         *         *         *         *         *
13351 AGGATTAGTTGGAATAAAAGAACAATAAGAACAGAATACACAATGCCTTGAGAAAGTTATTATTCAGATACTTACATTAAAACCCTTTTGGTAAGAAGTGCCTGGAGTTGCAGCCACTCCTTTATTTATATATTAACTATGAAATGATTA 13500
      ||||||||||||||||||||||||||||||||||||||||||||||||||||||||||||||||||||||||||||||||||||||||||||||||||||||||||||||||||||||||||||||||||||||||||||||||||||||
13345 AGGATTAGTTGGAATAAAAGAACAATAAGAACAGAATACACAATGCCTTGAGAAAGTTATTATTCAGATACTTACATTAAAACCCTTTTGGTAAGAAGTGCCTGGAGTTGCAGCCACTCCTTTATTTATATATTAACTATGAAATGATTA 13494
           *         *         *         *         *         *         *         *         *         *         *         *         *         *         *

               *         *         *         *         *         *         *         *         *         *         *         *         *         *         *
13501 TGTTTAAAACCAGAAAATCATGTTTATAATTTTTAAAATAAGCTATTCCAGGGTCACCTTGTAAAATTAAACTGATTCCAAGTCTTATCTGCCACAACAAAACATAAAGATGAATACACAAAAGAAGAAAGGATGTACAAAGGCAATAGC 13650
      ||||||||||||||||||||||||||||||||||||||||||||||||||||||||||||||||||||||||||||||||||||||||||||||||||||||||||||||||||||||||||||||||||||||||||||||||||||||
13495 TGTTTAAAACCAGAAAATCATGTTTATAATTTTTAAAATAAGCTATTCCAGGGTCACCTTGTAAAATTAAACTGATTCCAAGTCTTATCTGCCACAACAAAACATAAAGATGAATACACAAAAGAAGAAAGGATGTACAAAGGCAATAGC 13644
           *         *         *         *         *         *         *         *         *         *         *         *         *         *         *

               *         *         *         *         *         *         *         *         *         *         *         *         *         *         *
13651 ATTTCCATAATAGAGCAATTAAACTTTTAATTTACAGTAAACAAGGAGGTTTCAAAATGAACAGCCCTTTTGGGCAATTTTGCTAACGTAAGAAGAAAACTTACAGCGCTCTCCAGAACTGTATGCCAGAAACAAAAACGGGATGTACAA 13800
      ||||||||||||||||||||||||||||||||||||||||||||||||||||||||||||||||||||||||||||||||||||||||||||||||||||||||||||||||||||||||||||||||||||||||||||||||||||||
13645 ATTTCCATAATAGAGCAATTAAACTTTTAATTTACAGTAAACAAGGAGGTTTCAAAATGAACAGCCCTTTTGGGCAATTTTGCTAACGTAAGAAGAAAACTTACAGCGCTCTCCAGAACTGTATGCCAGAAACAAAAACGGGATGTACAA 13794
           *         *         *         *         *         *         *         *         *         *         *         *         *         *         *

               *         *         *         *         *         *         *         *         *         *         *         *         *         *         *
13801 ACCGTTTTACCCCCAAGGTTAAATGACATGAACATAAGATCAACCTGTCCCTAAACACTTACTCAAAATGCACCATAAAGAAATCTAGAAAATATTTCTTTTGGGGGGAATCAGGCGGCCGTGGATATCATCATCCATCCCAAAAATGGC 13950
      ||||||||||||||||||||||||||||||||||||||||||||||||||||||||||||||||||||||||||||||||||||||||||||||||||||||||||||||||||||||||||||||||||||||||||||||||||||||
13795 ACCGTTTTACCCCCAAGGTTAAATGACATGAACATAAGATCAACCTGTCCCTAAACACTTACTCAAAATGCACCATAAAGAAATCTAGAAAATATTTCTTTTGGGGGGAATCAGGCGGCCGTGGATATCATCATCCATCCCAAAAATGGC 13944
           *         *         *         *         *         *         *         *         *         *         *         *         *         *         *

               *         *         *         *         *         *         *         *         *         *         *         *         *         *         *
13951 ACTGGTTAGTAAGGCCAGCAAAAAGAGTTCTGCAACACGCATTCCTGTAGTGGTAGCCAATGAAGATGCAAAGGGTCGAGACCAAAAGGATATTAGTAGCTTGGTTTGACAGAATCATACCTGAAGGTTACCGCAACAAAACTACATTCA 14100
      ||||||||||||||||||||||||||||||||||||||||||||||||||||||||||||||||||||||||||||||||||||||||||||||||||||||||||||||||||||||||||||||||||||||||||||||||||||||
13945 ACTGGTTAGTAAGGCCAGCAAAAAGAGTTCTGCAACACGCATTCCTGTAGTGGTAGCCAATGAAGATGCAAAGGGTCGAGACCAAAAGGATATTAGTAGCTTGGTTTGACAGAATCATACCTGAAGGTTACCGCAACAAAACTACATTCA 14094
           *         *         *         *         *         *         *         *         *         *         *         *         *         *         *

               *         *         *         *         *         *         *         *         *         *         *         *         *         *         *
14101 GTTGTGCAATGGCGCAAAGAAAAACCAAAGCAGAAGCTAAGTTTTAGTAGAAGAGTGATCTATTATACAGTGTGGCATGTGTTAATCTGTGAAGCAGACTCCTGTTGATTTGGTAAACAGTTCATCTCAATTCTCCTGCTTCAGAGGTGA 14250
      ||||||||||||||||||||||||||||||||||||||||||||||||||||||||||||||||||||||||||||||||||||||||||||||||||||||||||||||||||||||||||||||||||||||||||||||||||||||
14095 GTTGTGCAATGGCGCAAAGAAAAACCAAAGCAGAAGCTAAGTTTTAGTAGAAGAGTGATCTATTATACAGTGTGGCATGTGTTAATCTGTGAAGCAGACTCCTGTTGATTTGGTAAACAGTTCATCTCAATTCTCCTGCTTCAGAGGTGA 14244
           *         *         *         *         *         *         *         *         *         *         *         *         *         *         *

               *         *         *         *
14251 AGATTTGATGATGCTCCAATCATCATTCTTATCATCCTCCCAGGT 14295
      |||||||||||||||||||||||||||||||||||||||||||||
14245 AGATTTGATGATGCTCCAATCATCATTCTTATCATCCTCCCAGGT 14289
           *         *         *         *
```
