# Supplementary material for: The major‐effect quantitative trait locus Fnl7.1 encodes a late embryogenesis abundant protein associated with fruit neck length in cucumber
Source: Plant Biotechnol J. 2020 Jan 24;18(7):1598–609. doi: 10.1111/pbi.13326 (PMC7292543; doi:10.1111/pbi.13326)

Fig. S2 Alignment of the promoter sequences of Jin5-508 (upper) and YN (lower).The pair-wise comparison was performed by using the ApE (v2.0.60) software with default parameters. The software is accessible from http://jorgensen.biology.utah.edu/wayned/ape/. The variants (SNPs and InDels) were highlighted in red.

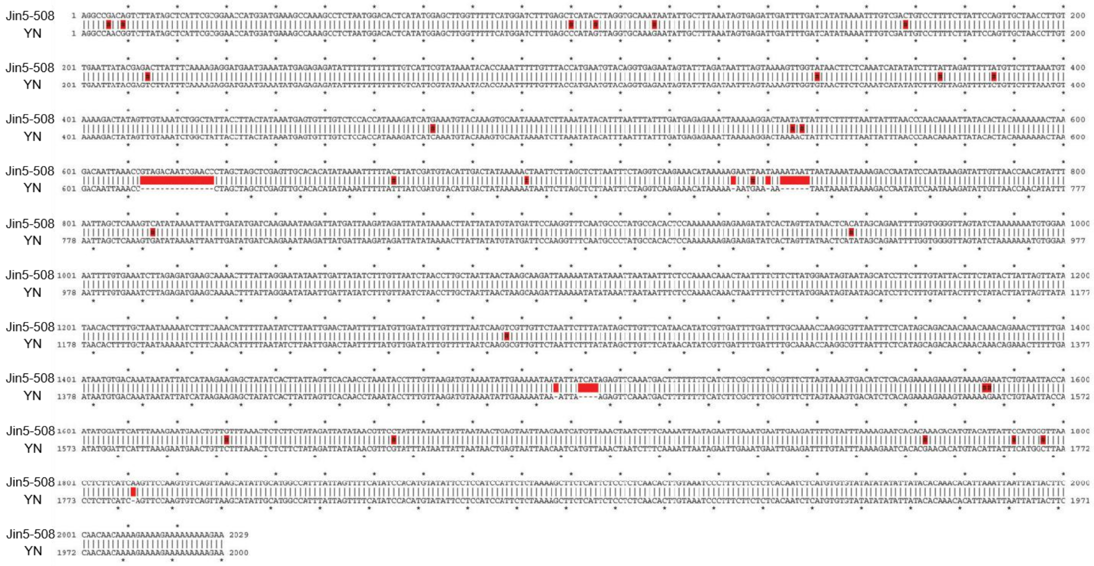

Supplement: Supplementary file 2 — Figure S2 Alignment of the promoter sequences of Jin5‐508 and YN. [file PBI-18-1598-s003.pdf]
